# Proper protein folding in the endoplasmic reticulum is required for attachment of a glycosylphosphatidylinositol anchor in plants

**DOI:** 10.1093/plphys/kiab181

**Published:** 2021-04-30

**Authors:** Yun-Ji Shin, Ulrike Vavra, Richard Strasser

**Affiliations:** Department of Applied Genetics and Cell Biology, University of Natural Resources and Life Sciences, Muthgasse 18, A-1190 Vienna, Austria

## Abstract

Endoplasmic reticulum (ER) quality control processes recognize and eliminate misfolded proteins to maintain cellular protein homeostasis and prevent the accumulation of defective proteins in the secretory pathway. Glycosylphosphatidylinositol (GPI)-anchored proteins carry a glycolipid modification, which provides an efficient ER export signal and potentially prevents the entry into ER-associated degradation (ERAD), which is one of the major pathways for clearance of terminally misfolded proteins from the ER. Here, we analyzed the degradation routes of different misfolded glycoproteins carrying a C-terminal GPI-attachment signal peptide in *Arabidopsis thaliana*. We found that a fusion protein consisting of the misfolded extracellular domain from Arabidopsis STRUBBELIG and the GPI-anchor attachment sequence of COBRA1 was efficiently targeted to hydroxymethylglutaryl reductase degradation protein 1 complex-mediated ERAD without the detectable attachment of a GPI anchor. Non-native variants of the GPI-anchored lipid transfer protein 1 (LTPG1) that lack a severely misfolded domain, on the other hand, are modified with a GPI anchor and targeted to the vacuole for degradation. Impaired processing of the GPI-anchoring signal peptide by mutation of the cleavage site or in a GPI-transamidase-compromised mutant caused ER retention and routed the non-native LTPG1 to ERAD. Collectively, these results indicate that for severely misfolded proteins, ER quality control processes are dominant over ER export. For less severely misfolded proteins, the GPI anchor provides an efficient ER export signal resulting in transport to the vacuole.

## Teaser

Severely misfolded proteins carrying a glycosylphosphatidylinositol (GPI)-anchor attachment sequence undergo a stringent quality control process in the endoplasmic reticulum that prevents GPI anchoring.

## Introduction

In eukaryotes, nearly one-third of all proteins are targeted to the endoplasmic reticulum (ER) where they undergo folding and maturation before the proteins are transported to other cellular sites (Ghaemmaghami et al., 2003). Protein folding is, however, prone to errors and newly synthesized proteins that do not pass ER quality control (ERQC) processes are typically targeted for ER-associated degradation (ERAD) to prevent their accumulation and the secretion of potentially deleterious non-native proteins (Vembar and Brodsky, 2008; Hüttner and Strasser, 2012; Liu and Howell, 2016; Chen et al., 2020). Anchoring of glycosylphosphatidylinositol (GPI) to proteins in the ER is a conserved posttranslational modification. In plants, more than 200 proteins are predicted to be modified with a GPI anchor (Borner et al., 2003; Elortza et al., 2006; Yeats et al., 2018; Desnoyer and Palanivelu, 2020) and in *Arabidopsis thaliana* loss of GPI-anchoring is embryo lethal (Gillmor et al., 2005). During GPI-anchoring, the GPI-transamidase complex cleaves off a hydrophobic C-terminal GPI-attachment signal peptide and transfers the preassembled GPI anchor to the newly formed carboxyl end of the protein. Thus, the GPI-anchor modification links the protein to the luminal leaflet of the lipid bilayer (Kinoshita and Fujita, 2016). In yeast and mammals, the GPI anchor acts as an ER-export signal, and GPI-anchored proteins are incorporated into Coat Protein Complex II (COPII) vesicles and traffic through the Golgi apparatus to the plasma membrane. For example, mammalian prion protein is GPI-anchored and misfolded variants of this protein are implicated in neurodegenerative diseases (Kovács et al., 2002). Previous studies indicate that such misfolded GPI-anchored proteins are not efficiently cleared by ERAD and instead routed via the secretory pathway to lysosomes for degradation (Ashok and Hegde, 2008, 2009; Satpute-Krishnan et al., 2014). The GPI anchor may provide a steric hindrance for membrane extraction, retrotranslocation into the cytosol or proteasomal degradation, and therefore misfolded GPI-anchored proteins could be poor substrates and degraded by an alternative pathway. Furthermore, the GPI anchor provides a strong ER export signal that competes with retention by ERQC processes. In budding yeast (*Saccharomyces cerevisiae*), a misfolded GPI-anchored variant of beta-1,3-glucanosyltransferase (GAS1) is primarily degraded independent of the Hydroxymethylglutaryl Reductase Degradation Protein 1 (HRD1) ERAD complex (Fujita et al., 2007; Goder and Melero, 2011) and subjected to vacuolar degradation, especially when the GPI anchor is remodeled to present an efficient ER export signal (Sikorska et al., 2016). These findings suggest that ERAD-independent processes in the vacuole or lysosome preferentially degrade misfolded GPI-anchored proteins in yeast and mammals. Interestingly, however, ERAD with proteasomal degradation is the favored disposal route for misfolded GPI-anchored proteins in the protozoan parasite *Trypanosoma brucei* (Tiengwe et al., 2018).

In plants, recent findings report that p24 family proteins play a role in ER export and the transport of GPI-anchored proteins to the plasma membrane (Bernat-Silvestre et al., 2020). Previously, it was also shown that a variant of the Fasciclin-like Arabinogalactan Protein 4 (FLA4) lacking the GPI-anchoring signal is primarily retained in the ER (Xue et al., 2017). While a crucial function of the GPI anchor for subcellular targeting is slowly emerging, less is currently known about the degradation of GPI-anchored proteins in plants.

Because of the apparent differences in degradation of misfolded GPI-anchored proteins between eukaryotic species and our lack of knowledge about this process in plants, we investigated the fate of misfolded GPI-anchored proteins. Previously, we showed that the misfolded extracellular domain from Arabidopsis STRUBBELIG (SUBEX-C57Y) is a canonical ERAD substrate. SUBEX-C57Y is degraded in a glycan-dependent manner involving the HRD1 ERAD complex components Suppressor/Enhancer of Lin-12-Like (SEL1L), the lectin Osteosarcoma 9 (OS9) and mannose trimming by the α-mannosidases MNS4 or MNS5 (Hüttner et al., 2014b; Shin et al., 2018). The misfolded SUBEX-C57Y domain carries three N-glycans and the presence of a single N-glycan with a distinct exposed mannose residue is necessary and sufficient for ERAD (Hüttner et al., 2014b). Notably, the same ERAD machinery degrades SUBEX-C57Y when attached to different transmembrane domains (Shin et al., 2018). Here, we attached different GPI-anchor attachment sequences to SUBEX-C57Y and other misfolded proteins and analyzed their fate to understand the degradation pathway for defective GPI-anchored proteins. In contrast to other eukaryotes, our data show that ERQC and ERAD are very effective and rapidly degrade folding-defective proteins before the attachment of a GPI anchor. This suggests that GPI anchoring is a late posttranslational modification in plants when the protein has undergone folding and passed ERQC processes.

## Results

### A misfolded ERAD substrate fused to a GPI-anchor attachment sequence is subjected to HRD1 complex-mediated degradation

**Figure 1:**
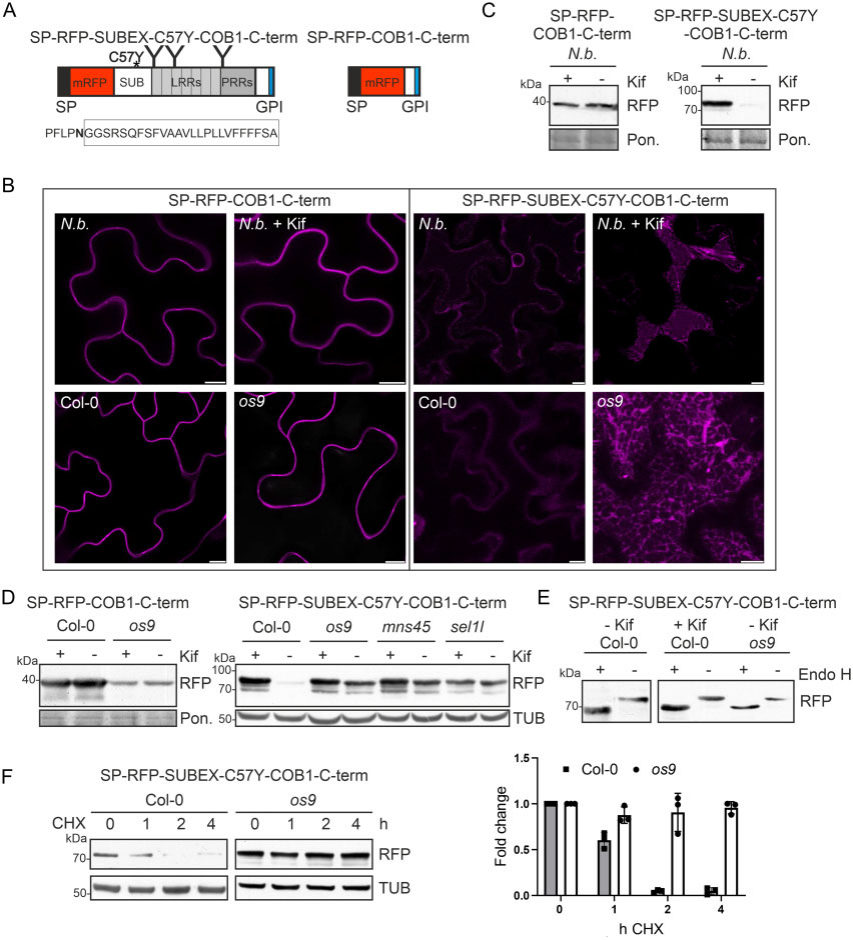
An ERAD substrate carrying a GPI-anchor attachment sequence is sent to glycan-dependent ERAD. A, Schematic illustration of SP-RFP-SUBEX-C57Y-COB1-C-term and SP-RFP-COB1-C-term proteins. SP: signal peptide; GPI: GPI-anchor attachment sequence; C57Y: amino acid change that leads to misfolding; SUB: SUB domain; LRRs: leucine-rich repeats; PRRs: proline-rich repeats; “Y”: N-glycans. The amino acid sequence of the COB1 C-terminus (residues 432–456 from Arabidopsis COBRA1) is shown. The predicted omega-site for attachment of the GPI-anchor is highlighted in bold and the hydrophobic C-terminal end is boxed. B, Confocal images of *N. benthamiana* (*N.b.*) leaf epidermal cells (upper panel) or Arabidopsis seedlings (Col-0 and the *os9* mutant, lower) expressing SP-RFP-SUBEX-C57Y-COB1-C-term or SP-RFP-COB1-C-term. The localization in the presence of kifunensine (Kif) is indicated. Scale bar = 10 µm. C, SP-RFP-COB1-C-term and SP-RFP-SUBEX-C57Y-COB1-C-term were transiently expressed in *N. benthamiana* in the presence or absence of Kif and at 2 d after infiltration were subjected to SDS–PAGE and immunoblotting using anti-RFP antibodies. Staining of membranes with Ponceau S (Pon.) was used as a loading control. D, Immunoblot analysis of transgenic wild-type (Col-0), *os9*, *mns45*, and *sel1l* plants upon incubation for 24 h in the presence of Kif. Staining of membranes with Ponceau S (Pon.) or detection of TUB was used as a loading control. E, Endo H digestion of SP-RFP-SUBEX-C57Y-COB1-C-term expressed in Arabidopsis Col-0 or *os9* plants in the absence or presence of Kif. Note that more protein was loaded and the film of the “Col-0 – Kif” panel was overexposed to make the bands visible. F, CHX treatment of Col-0 and *os9* seedlings. The fold change relative to the starting amounts of SP-RFP-SUBEX-C57Y-COB1-C-term was calculated after normalization to TUB. Error bars indicate means ± sd (*n* = 3).

Given that misfolded GPI-anchored proteins are poor ERAD substrates in mammals and yeast (Ashok and Hegde, 2008; Ashok and Hegde, 2009; Satpute-Krishnan et al., 2014; Sikorska et al., 2016), we examined the fate of a misfolded glycoprotein carrying a GPI-anchor attachment sequence in plants. To this end, SUBEX-C57Y, the misfolded extracellular domain from the receptor-like kinase STRUBBELIG (without the native signal peptide, amino acids 25–341; Hüttner et al., 2014b; Shin et al., 2018) was fused to the GPI-anchoring region from Arabidopsis COBRA1 (COB1; Schindelman et al., 2001; Roudier et al., 2005) and to mRFP carrying an N-terminal signal peptide to generate a misfolded GPI-anchored protein termed SP-RFP-SUBEX-C57Y-COB1-C-term. For comparison, we generated a variant lacking the folding-defective SUBEX-C57Y domain (SP-RFP-COB1-C-term; Figure 1A). In silico prediction of the GPI-anchor signal sequences using the big-PI Plant Predictor tool (Eisenhaber et al., 2003) revealed similar scores for the predicted site for GPI-anchor attachment (Supplemental Figure S1), suggesting that SP-RFP-SUBEX-C57Y-COB1-C-term and SP-RFP-COB1-C-term are both GPI-anchored proteins. When transiently expressed in *Nicotiana benthamiana*, SP-RFP-COB1-C-term was localized at the plasma membrane (Figure 1B) as previously shown for other GPI-anchored proteins (Zavaliev et al., 2016) and a band of expected size was detected on immunoblots (Figure 1C). In contrast, SP-RFP-SUBEX-C57Y-COB1-C-term accumulated only in the presence of the ERAD inhibitor kifunensine that blocks ERAD by interference with the formation of the glycan signal required for degradation (Figure 1C; Hüttner et al., 2014a). In *N. benthamiana* leaf epidermal cells, SP-RFP-SUBEX-C57Y-COB1-C-term was barely visible under the confocal microscope. The few cells with faint fluorescence displayed primarily ER localization. In the presence of kifunensine, the fluorescence signal was increased and more cells with ER localization of SP-RFP-SUBEX-C57Y-COB1-C-term were found (Figure 1B, top). Similarly, in Arabidopsis wild-type expressing SP-RFP-SUBEX-C57Y-COB1-C-term, only a faint fluorescence signal was detected. When Col-0 was treated with kifunensine or in different ERAD mutants with a blocked ERAD pathway (*os9*, *mns45*, or *sel1l*; Hüttner et al., 2012, 2014a), SP-RFP-SUBEX-C57Y-COB1-C-term displayed primarily ER labeling in leaves (Figure 1B, bottom) and in roots (Supplemental Figure S2). Immunoblot analysis confirmed that SP-RFP-SUBEX-C57Y-COB1-C-term is hardly detectable in Arabidopsis wild-type, but the protein accumulated in the presence of kifunensine or in mutants, which have a dysfunctional glycan-dependent ERAD pathway (Figure 1D). SP-RFP-COB1-C-term was not affected in a similar manner in the wild-type (Figure 1B) or when ERAD was blocked (Figure 1D) and displayed a steady-state localization at the plasma membrane in leaves (Figure 1B) and roots (Supplemental Figure S2).

The SUBEX-C57Y domain carries three N-glycosylation sites and ER retention of a variant lacking a membrane anchoring region results in the presence of endoglycosidase H (Endo H) sensitive oligomannosidic N-glycans (Hüttner et al., 2014b). Consistent with the ER localization, SP-RFP-SUBEX-C57Y-COB1-C-term carried exclusively Endo H sensitive N-glycans in the wild-type as well as in the *os9* mutant with a defective ERAD pathway (Figure 1E). To monitor the protein stability over time, we inhibited the protein synthesis with cycloheximide (CHX). Immunoblots revealed that SP-RFP-SUBEX-C57Y-COB1-C-term was degraded much faster in the wild-type compared to the *os9* mutant (Figure 1F). Taken together, these data define SP-RFP-SUBEX-C57Y-COB1-C-term as a canonical ERAD substrate that is subjected to glycan-dependent degradation in the ER.

### SP-RFP-SUBEX-C57Y-COB1-C-term lacks a GPI anchor

**Figure 2:**
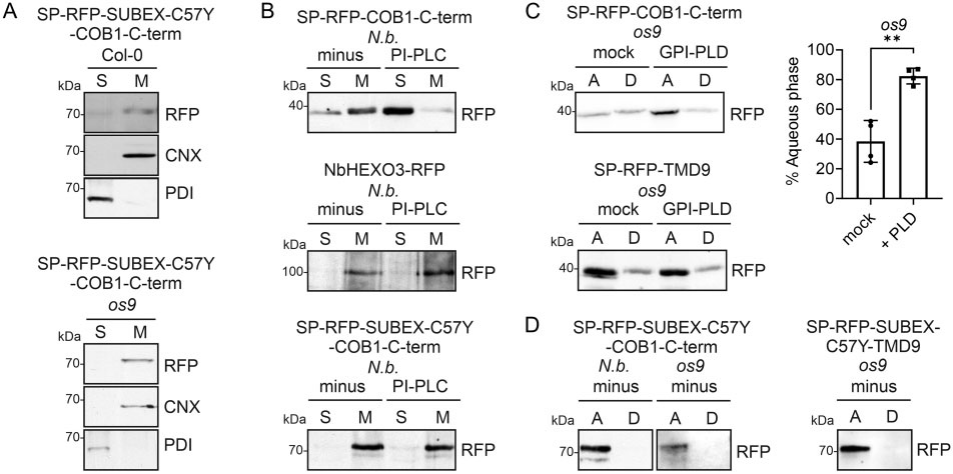
SP-RFP-SUBEX-C57Y-COB1-C-term is not GPI-anchored. A, Distribution of SP-RFP-SUBEX-C57Y-COB1-C-term in soluble (S) and membrane (M) fractions of Col-0 and *os9* seedlings. Immunoblot analysis was carried out with anti-RFP (RFP), anti-CNX, and anti-protein disulphide isomerase antibodies. B, PI-PLC digestion of membrane (M) fractions from transiently expressed SP-RFP-COB1-C-term, NbHEXO3-RFP and SP-RFP-SUBEX-C57Y-COB1-C-term. C, Microsomal fractions from the Arabidopsis *os9* mutant were solubilized with 0.1% NP-40, GPI-PLD digested and subjected to Triton X-114 extraction to separate the aqueous (A) and detergent (D) phases. “mock” indicates incubation with a control cell extract expressing a truncated inactive GPI-PLD. SP-RFP-TMD9 that contains a transmembrane domain (TMD9) instead of the GPI-anchor attachment sequence was included as a control. Histogram data: relative amount of SP-RFP-COB1-C-term in the aqueous phase; Error bars indicate mean ± sd (*n* = 3, “**” *P* <0.01 according to a Student’s *t* test). D, Triton X-114 phase separation of SP-RFP-SUBEX-C57Y-COB1-C-term expressed in *N. benthamiana* or the Arabidopsis *os9* mutant. SP-RFP-SUBEX-C57Y-TMD9 that contains a transmembrane domain (TMD9) instead of the GPI-anchor attachment sequence was included as a control. “Minus” indicates that no digestion with GPI-PLD was carried out.

To test the possibility that ERAD is initiated after GPI anchoring, we examined the modification of SP-RFP-SUBEX-C57Y-COB1-C-term with a GPI anchor. Membrane and soluble proteins were separated from each other and subjected to immunoblotting to examine whether SP-RFP-SUBEX-C57Y-COB1-C-term is membrane-bound. In Col-0 as well as in the *os9* mutant where SP-RFP-SUBEX-C57Y-COB1-C-term can readily be detected on immunoblots, SP-RFP-SUBEX-C57Y-COB1-C-term was present in the same fraction as the ER-resident membrane-bound calnexin (CNX; Figure 2A). Next, we digested microsomal fractions with phosphatidylinositol-phospholipase C (PI-PLC) to release GPI-anchored proteins from the membrane into the soluble fraction (Roudier et al., 2005; Sikorska et al., 2016; Gong et al., 2017; Liu et al., 2018). In the undigested control, the majority of SP-RFP-COB1-C-term appeared in the membrane-containing fraction. Upon digestion with PI-PLC, SP-RFP-COB1-C-term was mainly recovered in the soluble fraction (Figure 2B, top) showing that SP-RFP-COB1-C-term is GPI-anchored to the membrane. This is consistent with the recent mass spectrometry (MS)-based detection of a GPI anchor on SP-RFP-COB1-C-term (Beihammer et al., 2020). The plasma membrane protein β-hexosaminidase 3 (NbHEXO3-mRFP), which carries an N-terminal transmembrane domain (Shin et al., 2017), was used as a control and was not detected in the supernatant after PI-PLC digestion (Figure 2B, middle). In contrast to SP-RFP-COB1-C-term, SP-RFP-SUBEX-C57Y-COB1-C-term was completely resistant to PI-PLC treatment (Figure 2B, bottom) and found in the membrane fraction.

Upon transfer to the protein, the GPI anchor undergoes remodeling, which includes inositol-deacylation catalyzed by the GPI-inositol deacylase Post-GPI attachment to protein 1 (Kinoshita and Fujita, 2016). Acylation of myo-inositol blocks PI-PLC cleavage of GPI anchors (Liu et al., 2018). Although GPI anchors appear remodeled irrespective of the protein folding status in yeast cells (Sikorska et al., 2016), it is plausible that the ER-retained SP-RFP-SUBEX-C57Y-COB1-C-term is still inositol-acylated, which may explain the absence in the soluble fraction upon PI-PLC digestion. Human phospholipase D (GPI-PLD) is not blocked by the presence of GPI-anchor modifications such as acylation and can be used instead of PI-PLC to release the GPI anchor from a protein. However, while PI-PLC attacks GPI-anchored proteins in membranes, GPI-PLD is poorly active on GPI-anchored proteins from intact membranes (Low and Huang, 1991; Deeg and Davitz, 1995). Therefore, to establish a procedure suitable for GPI-PLD digestion, microsomal membrane fractions containing the GPI-anchored protein SP-RFP-COB1-C-term were incubated with PI-PLC or GPI-PLD in the presence of 0.1% of the nonionic detergent NP-40. The aqueous and detergent phase was subsequently isolated using a Triton X-114 two-phase separation step (Bordier, 1981). In line with the presence of a GPI anchor, PI-PLC digestion caused the complete removal of SP-RFP-COB1-C-term from the detergent phase (Supplemental Figure S3). Addition of mammalian cell culture supernatant expressing recombinant GPI-PLD resulted in a significant increase of SP-RFP-COB1-C-term in the aqueous phase compared to the incubation with a cell culture supernatant expressing an inactive GPI-PLD fragment (“mock”) as a control (Figure 2C).

Next, we carried out the microsomal membrane preparation, NP-40 solubilization and Triton X-114 two-phase separation with SP-RFP-SUBEX-C57Y-COB1-C-term to test whether GPI-PLD can release the ER-retained protein. However, SP-RFP-SUBEX-C57Y-COB1-C-term was exclusively found in the aqueous phase in the absence of GPI-PLD (Figure 2D). SP-RFP-SUBEX-C57Y-COB1-C-term essentially behaved as SP-RFP-SUBEX-C57Y-TMD9 carrying a C-terminal transmembrane domain (Shin et al., 2018) instead of the COB1 GPI-anchor attachment sequence (Figure 2D). Taken together, these findings suggest that ER-retained SP-RFP-SUBEX-C57Y-COB1-C-term does not carry a GPI anchor but instead retains the C-terminal hydrophobic signal sequence that serves as a transmembrane domain and is inserted into the ER membrane before being transferred to a GPI anchor (Kinoshita and Fujita, 2016).

**Figure 3:**
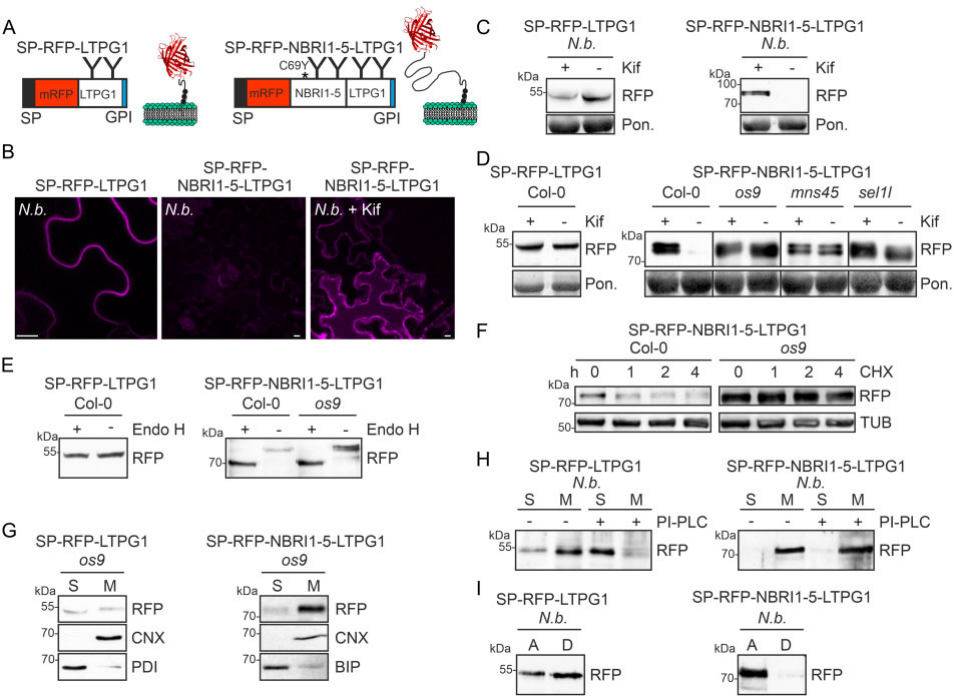
SP-RFP-NBRI1-5-LTPG1 is subjected to glycan-dependent ERAD, but is not GPI-anchored. A, Schematic illustration of SP-RFP-LTPG1 and SP-RFP-NBRI1-5-LTPG1. SP: signal peptide; C69Y: amino acid change that causes the misfolding of the N-terminal BRI1 domain (NBRI1-5); GPI: GPI-anchor attachment sequence; “Y”: N-glycans. B, Confocal images of SP-RFP-LTPG1 and SP-RFP-NBRI1-5-LTPG1 transiently expressed in *N. benthamiana* (*N.b.*) in the absence or presence of kifunensine (Kif). Scale bars = 10 µm. C, Immunoblot analysis of transiently expressed SP-RFP-LTPG1 and SP-RFP-NBRI1-5-LTPG1 in the absence or presence of Kif. Staining of membranes with Ponceau S (Pon.) was used as a loading control. D, Immunoblot analysis of stably expressed SP-RFP-LTPG1 or SP-RFP-NBRI1-5-LTPG1. E, Endo H digestion of SP-RFP-LTPG1 and SP-RFP-NBRI1-5-LTPG1 expressed in Arabidopsis wild-type (Col-0) or the *os9* mutant. F, CHX-treatment of Col-0 and *os9* seedlings. G, Distribution in soluble (S) and membrane (M) fractions of *os9* or *sel1l* seedlings. H, Immunoblot analysis of PI-PLC-digested membrane (M) fractions from *N. benthamiana* expressing SP-RFP-LTPG1 or SP-RFP-NBRI1-5-LTPG1 and (I) of 0.1% NP-40 solubilized and Triton X-114 phase-separated aqueous (A) and detergent (D) fractions.

To examine how other GPI-anchor attachment sequences behave when attached to defective proteins, we fused the N-terminal portion without the native signal peptide (amino acids 24–212) of the misfolded brassinosteroid receptor BRASSINOSTEROID INSENSITIVE 1–5 (called NBRI1-5; Hong et al., 2008; Shin et al., 2018) to the lipid transfer protein LTPG1 from Arabidopsis, which was previously identified as a GPI-anchored protein (Borner et al., 2003; Lee et al., 2009; Figure 3A). The misfolded BRI1 domain in NBRI1-5 carries a cysteine to tyrosine exchange at position 69 (C69Y), which disrupts a disulfide bond (Hong et al., 2008). Using confocal microscopy, the expression of SP-RFP-NBRI1-5-LTPG1 was barely detectable (Figure 3B). However, the protein accumulated in the ER and vacuole in the presence of kifunensine (Figure 3B) and its degradation was blocked in *N. benthamiana* (Figure 3C) and in Arabidopsis mutants with a dysfunctional ERAD pathway (Figure 3D). Moreover, SP-RFP-NBRI1-5-LTPG1 carried Endo H sensitive N-glycans (Figure 3E) and was cleared faster in the wild-type compared to the *os9* mutant, showing that it is a glycan-dependent ERAD substrate (Figure 3F). In contrast, SP-RFP-LTPG1 lacking the misfolded domain labeled the plasma membrane (Figure 3B), accumulated in the wild-type (Figure 3D) and displayed Endo H-resistant glycans (Figure 3E).

While considerable amounts of SP-RFP-LTPG1 and SP-RFP-NBRI1-5-LTPG1 were detected in the membrane-containing fraction (Figure 3G), following PI-PLC treatment, an increase of the protein in the soluble fraction could only be observed for SP-RFP-LTPG1 (Figure 3H). Moreover, upon Triton X-114 extraction SP-RFP-NBRI1-5-LTPG1 was exclusively present in the aqueous phase (A) without phospholipase digestion (Figure 3I). Essentially, the same result was obtained with a SP-RFP-SUBEX-C57Y-LTPG1 fusion protein (Supplemental Figure S4). Taken together, the characterization of different glycosylated ERAD substrates carrying GPI-anchor attachment sequences revealed no evidence that these proteins carry a lipid anchor.

### A cysteine-deficient LTPG1 variant is not subjected to glycan-dependent ERAD

**Figure 4:**
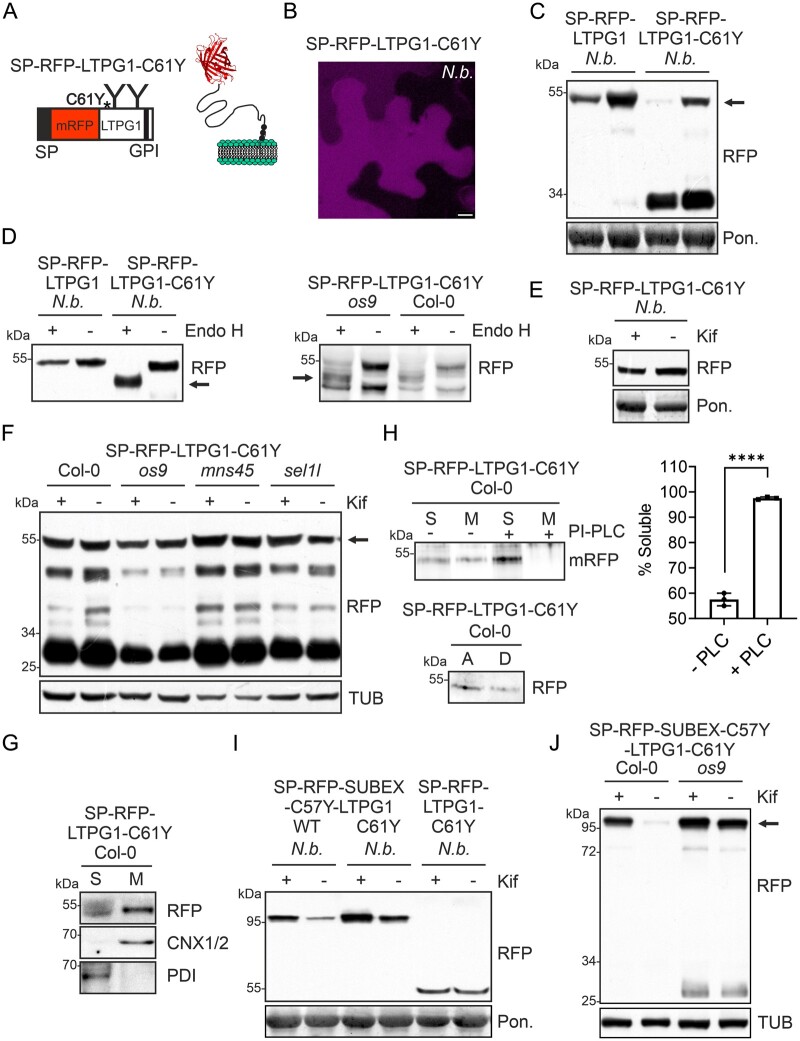
Cysteine-deficient SP-RFP-LTPG1-C61Y is not subjected to ERAD. A, Schematic illustration of SP-RFP-LTPG1-C61Y. SP: signal peptide; C61Y: amino acid change; GPI: GPI-anchor attachment sequence; “Y”: N-glycans. B, Confocal image of transiently expressed SP-RFP-LTPG1-C61Y. Scale bar = 10 µm. C, Immunoblot analysis of transiently expressed proteins. SP-RFP-LTPG1 and SP-RFP-LTPG1-C61Y were infiltrated with two different ODs (0.05 and 0.2) into *N. benthamiana* (*N.b.*) leaves. The position of the intact fusion protein is indicated by an arrow. Staining of membranes with Ponceau S (Pon.) was used as a loading control. D, Endo H digestion and immunoblot analysis of transiently (*N.b.*) or stably (Col-0, *os9*) expressed proteins. The arrow marks the specific band after Endo H cleavage. E, Immunoblot analysis of SP-RFP-LTPG1-C61Y transiently expressed with kifunensine (Kif). F, Immunoblot analysis of stably expressed SP-RFP-LTPG1-C61Y in the presence of Kif. G, Distribution of SP-RFP-LTPG1-C61Y in soluble (S) and membrane (M) fractions. H, Immunoblot analysis of PI-PLC-digested membrane (M) and soluble (S) fractions and Triton X-114 phase-separated aqueous (A) and detergent (D) fractions. The relative amount of SP-RFP-LTPG1-C61Y in the soluble (S) fraction was quantified. The data represent mean ± sd (*n* = 3, “****” *P* <0.0001 according to a Student’s *t* test). I, Immunoblot analysis of SP-RFP-SUBEX-C57Y-LTPG1, SP-RFP-SUBEX-C57Y-LTPG1-C61Y, and SP-RFP-LTPG1-C61Y transiently expressed in *N. benthamiana* with Kif. J, Immunoblot analysis of stably expressed SP-RFP-SUBEX-C57Y-LTPG1-C61Y.

In the previous experiments, we showed that glycoprotein ERAD substrates such as SUBEX-C57Y or NBRI1-5 carrying a GPI-anchor attachment sequence are retained in the ER and cleared by ERAD without the attachment of a GPI anchor. Next, we examined the fate of an aberrant GPI-anchored protein lacking a known ERAD-mediating domain. As a structurally compromised GPI-anchored protein, we generated a mutant variant of LTPG1 where a conserved cysteine residue is changed to a tyrosine (C61Y; Lee et al., 2009). Due to the introduced amino acid exchange, LTPG1-C61Y is likely misfolded and recognized by a thiol-based ER retention mechanism similar to the retention and degradation of the misfolded BRI1-5 receptor (Hong et al., 2008). LTPG1-C61Y harbors two potential N-glycosylation sites (N110 and N135) that may facilitate clearance by the glycan-dependent ERAD pathway (Figure 4A). When SP-RFP-LTPG1-C61Y was transiently expressed in *N. benthamiana*, it was predominately found in the vacuole (compare Figure 4B to 3B). Immunoblot analysis revealed that the protein levels of the intact fusion protein were strongly reduced compared to SP-RFP-LTPG1 (Figure 4C), and considerable amounts of cleaved RFP were detectable for SP-RFP-LTPG1-C61Y. In contrast to SP-RFP-LTPG1, SP-RFP-LTPG1-C61Y was sensitive to Endo H digestion (Figure 4D), indicating that the protein carries oligomannosidic N-glycans that are not processed in the Golgi apparatus. Incomplete digestion of SP-RFP-LTPG1-C61Y with Endo H confirmed further that both N-glycosylation sites carry N-glycans and can be fully de-glycosylated (Supplemental Figure S5). Co-infiltration of *N. benthamiana* leaves with the ERAD inhibitor kifunensine did not result in accumulation of SP-RFP-LTPG1-C61Y on immunoblots (Figure 4E). To validate this finding, we expressed SP-RFP-LTPG1-C61Y in Arabidopsis wild-type as well as in mutants with a defective ERAD pathway. Consistent with the data from transiently expressed SP-RFP-LTPG1-C61Y, no glycan-dependent degradation was detected in stable transformed Arabidopsis (Figure 4F). SP-RFP-LTPG1-C61Y was mainly membrane-anchored (Figure 4G) and digestion with PI-PLC as well as Triton X-114 separation indicated that SP-RFP-LTPG1-C61Y is GPI-anchored (Figure 4H). Next, we inserted the SUBEX-C57Y domain between RFP and LTPG1-C61Y to see if the presence of the ERAD-mediating domain in SP-RFP-SUBEX-C57Y-LTPG1-C61Y sends the protein to glycan-dependent ERAD. Transient (Figure 4I) and stable expression (Figure 4J) showed that SP-RFP-SUBEX-C57Y-LTPG1-C61Y was degraded by the glycan-dependent ERAD pathway.

It was unexpected to find that a LTPG1 variant with a single amino acid substitution at a conserved site is not subjected to glycan-dependent ERAD as observed for the LTPG1 variants fused to an ERAD-mediating misfolded domain such as NBRI1-5 (Figure 3) or SUBEX-C57Y (Supplemental Figure S4). To corroborate the finding, we expressed another LTPG1 variant, SP-RFP-LTPG1-C116Y, where the conserved cysteine at position 116 was changed to tyrosine (C116Y; Lee et al., 2009). SP-RFP-LTPG1-C116Y behaved in the same manner as the SP-RFP-LTPI-C61Y variant, i.e. were fully de-glycosylated by Endo H, and no glycan-dependent degradation was detected for SP-RFP-LTPG1-C116Y (Supplemental Figure S6). Taken together, these data demonstrate that a glycan-dependent ERAD pathway does not degrade aberrant LTPG1 variants with mutated cysteine residues unless they contain an additional misfolded domain.

### Aberrant LTPG1 with a mutated omega cleavage site is directed to glycan-dependent ERAD

**Figure 5:**
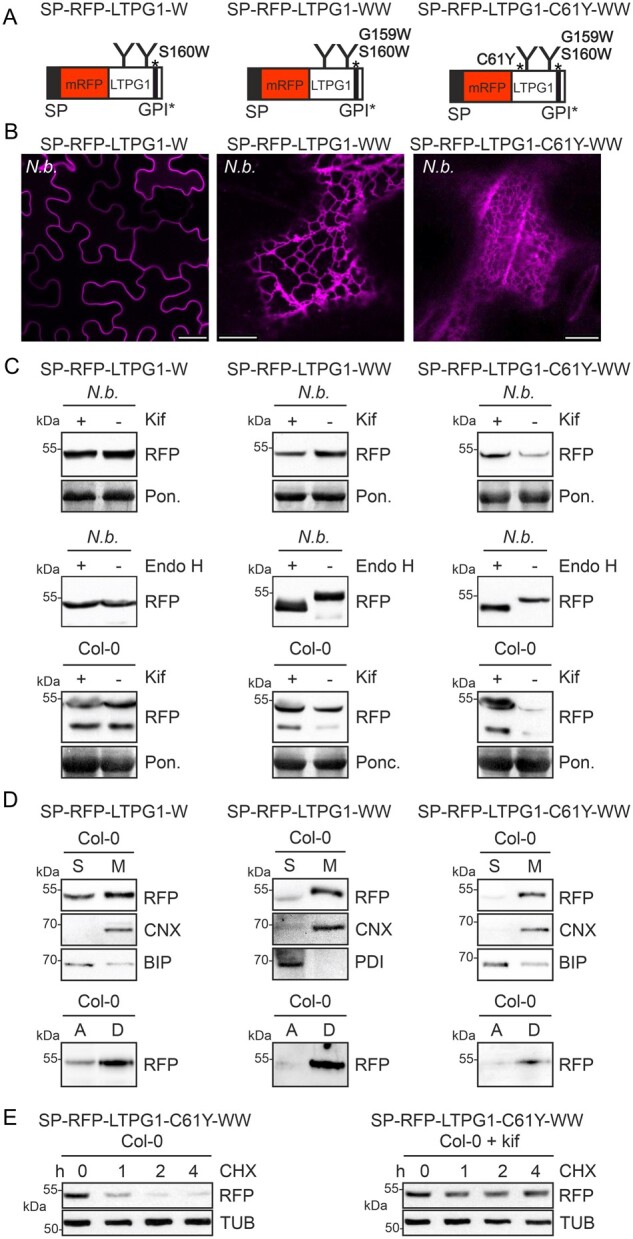
SP-RFP-LTPG1-C61Y with a defective omega cleavage site is directed to ERAD. A, Schematic illustration of SP-RFP-LTPG1-W, SP-RFP-LTPG1-WW, and SP-RFP-LTPG1-C61Y-WW. SP: signal peptide; C61Y, G159W and S160W amino acid changes; GPI: GPI-anchor attachment sequence; “Y”: N-glycans. B, Confocal images of transiently expressed proteins. Scale bars: SP-RFP-LTPG1-W = 25 µm, SP-RFP-LTPG1-WW, and SP-RFP-LTPG1-C61Y-WW = 10 µm. C, Immunoblot analysis of transiently or stably expressed proteins. Staining of membranes with Ponceau S (Pon.) was used as a loading control. D, Distribution of SP-RFP-LTPG1-W, SP-RFP-LTPG1-WW, and SP-RFP-LTPG1-C61Y-WW in soluble (S) and membrane (M) fractions and Triton X-114 phase separation. E, CHX treatment to monitor SP-RFP-LTPG1-C61Y-WW degradation over time.

In a previous study in mammalian cells, it was shown that impaired cleavage of the GPI-anchor attachment sequence targets proteins to ERAD (Ashok and Hegde, 2008). To examine how GPI-anchored proteins are affected in plants, we mutated the predicted omega site of LTPG1 (serine at position 160 changed to tryptophan, Supplemental Figure S7) and expressed SP-RFP-LTPG1-W transiently in *N. benthamiana* (Figure 5A). The protein was localized at the plasma membrane (Figure 5B), not affected by kifunensine and the N-glycans were resistant to Endo H digestion, indicating trafficking trough the Golgi apparatus (Figure 5C). Overall, this variant behaved like SP-RFP-LTPG1, suggesting that the S160W mutation neither abrogates GPI-anchoring nor leads to misfolding (Figure 5D). The plant big-PI Plant Predictor tool suggested that glycine at position 159 is an alternative cleavage site (Supplemental Figure S7). To examine this possibility, we expressed the SP-RFP-LTPG1-WW double mutant in *N. benthamiana*. SP-RFP-LTPG1-WW accumulated in the ER (Figure 5B) and the N-glycans were Endo H sensitive (Figure 5C). The steady-state protein levels were not affected by kifunensine when transiently expressed and only a minor increase was observed in Arabidopsis, suggesting that SP-RFP-LTPG1-WW is not efficiently subjected to glycan-dependent ERAD. In this scenario, the C-terminal transmembrane domain with the mutated GPI-anchor attachment sequence is not cleaved and the resulting protein stays in the ER because it lacks an ER exit signal. Due to the lack of a misfolded domain, it is not recognized as an ERAD substrate. Interestingly, however, the protein remained membrane-anchored via its C-terminal unprocessed domain (Figure 5D top) because the hydrophobic tryptophan residues likely contribute to anchoring and render it dependent on detergent solubilization.

When we introduced the C61Y lesion into SP-RFP-LTPG1-WW, SP-RFP-LTPG1-C61Y-WW localized in the ER (Figure 5B) and displayed Endo H-sensitive N-glycans (Figure 5C, center). However, in contrast to SP-RFP-LTPG1-WW and SP-RFP-LTPG1-C61Y, SP-RFP-LTPG1-C61Y-WW was clearly subjected to glycan-dependent ERAD in *N. benthamiana* (Figure 5C, top) and Arabidopsis (Figure 5C, bottom and Figure 5E). Like SP-RFP-LTPG1-WW, SP-RFP-LTPG1-C61Y-WW was mainly detected in the detergent phase (Figure 5D, bottom). These data indicate that structurally compromised glycoproteins with an additionally defective GPI-signal processing site are degraded by a glycan-dependent ERAD pathway.

### A dysfunctional GPI-transamidase causes ER retention and subsequent degradation by a glycan-dependent ERAD pathway of a structurally compromised glycoprotein

**Figure 6:**
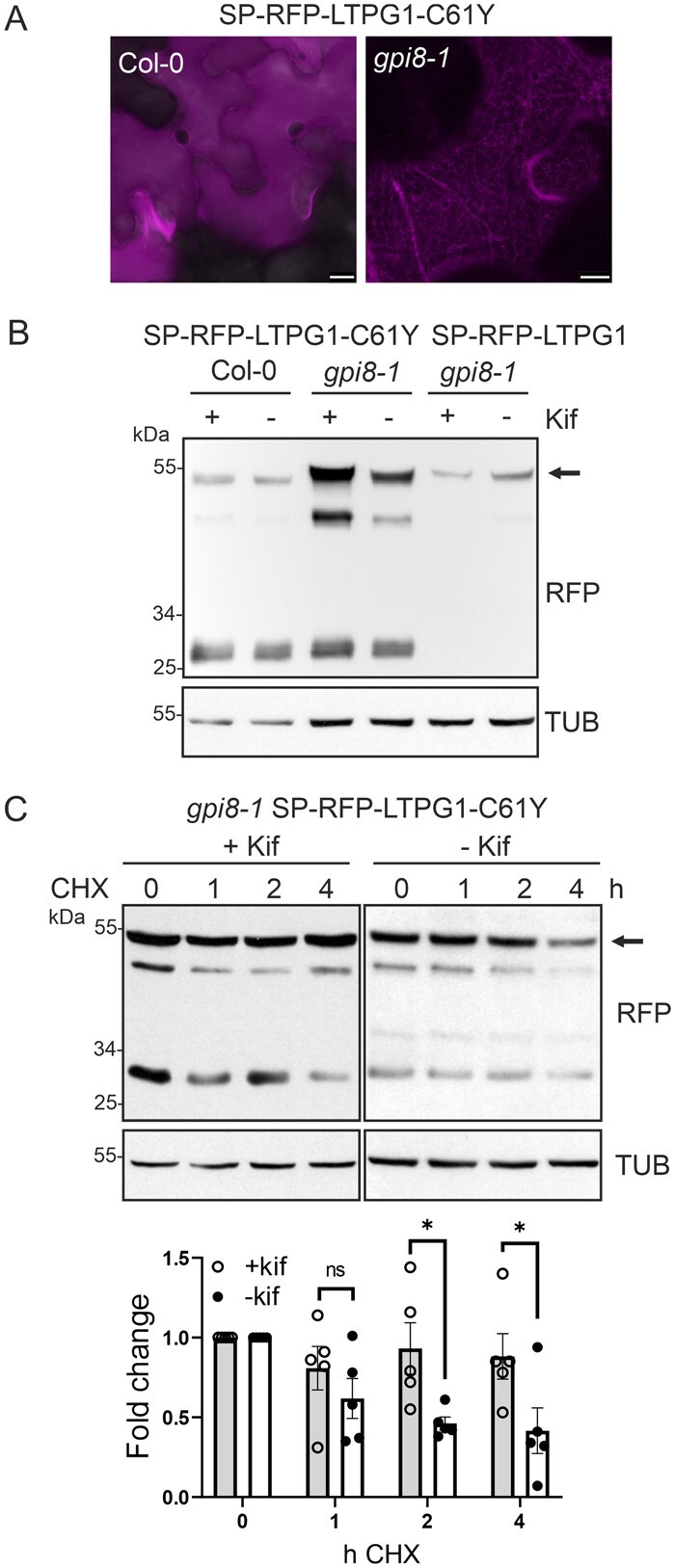
SP-RFP-LTPG1-C61Y is subjected to ERAD when GPI-anchoring is abolished. A, Confocal images of Arabidopsis shoot cells expressing SP-RFP-LTPG1-C61Y. Scale bars = 10 µm. B, Immunoblot analysis of stably expressed SP-RFP-LTPG1 and SP-RFP-LTPG1-C61Y in the absence or presence of kifunensine (Kif). C, CHX-treatment of *gpi8-1* seedlings expressing SP-RFP-LTPG1-C61Y. The fold change relative to the starting amount of SP-RFP-LTPG1-C61Y was calculated after normalization to TUB. Error bars indicate means ± sd (*n* = 5, “ns” not significant; “*” *P* < 0.05 according to a Student’s *t* test).

Recently, reduced protein levels of the GPI-anchored protein Monocopper oxidase-like protein fused to GFP (GFP-SKU5) were described in the Arabidopsis *gpi8-1* line, a partial-loss-of-function mutant carrying a missense mutation in the plant homolog of the GPI-transamidase subunit GPI8 (Bundy et al., 2016). The GPI-transamidase multi-protein complex is involved in the transfer of the assembled GPI anchor to proteins and it is anticipated that the transfer is reduced in the *gpi8-1* mutant. We next examined whether the aberrant SP-RFP-LTPG1-C61Y is subjected to ERAD in the *gpi8-1* mutant. In contrast to Col-0, SP-RFP-LTPG1-C61Y was detectable in the ER in the *gpi8-1* mutant (Figure 6A) and SP-RFP-LTPG1-C61Y was stabilized by kifunensine (Figure 6B). To confirm the stabilizing effect of kifunensine in the *gpi8-1* mutant, we followed the turnover of SP-RFP-LTPG1-C61Y over time (Figure 6C). An increase in the protein levels was detected at all time points in the presence of kifunensine. In contrast, no stabilizing effect of kifunensine was found in wild-type seedlings (Supplemental Figure S8). Together, these data show that SP-RFP-LTPG1-C61Y is retained in the ER and subjected to ERAD when the transfer of the glycoprotein to a GPI anchor is abolished in the g*pi8-1* mutant. In addition, the presence of cleaved RFP suggests that there is an additional degradation route involved, possibly by degradation in the vacuole. This route may be utilized by GPI-anchored variants that are still present in the leaky *gpi8-1* mutant.

### Under global stress conditions structurally compromised glycoproteins are subjected to glycan-dependent ERAD

**Figure 7:**
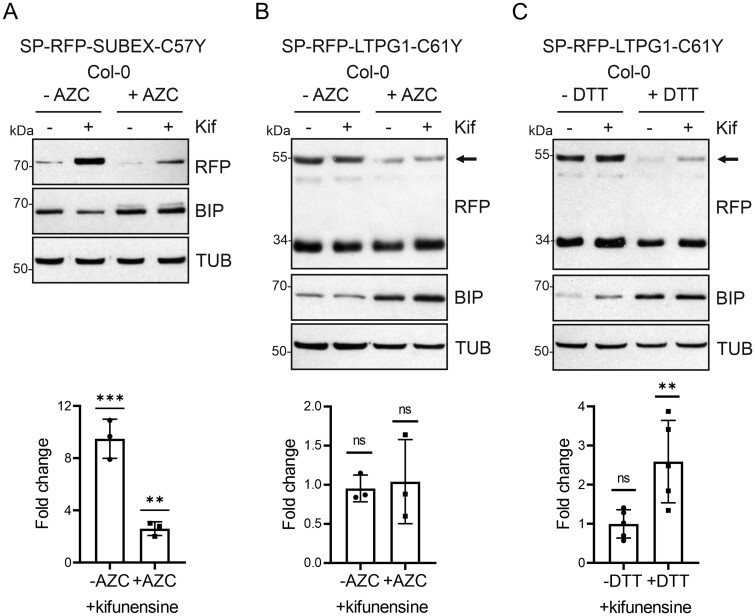
SP-RFP-LTPG1-C61Y is subjected to ERAD under global ER-stress conditions. A–C, Transgenic Arabidopsis Col-0 seedlings expressing SP-RFP-SUBEX-C57Y or SP-RFP-LTPG1-C61Y were incubated for 18 h in 5 mM AZC or 2 mM DTT to induce ER stress and subsequently subjected to immunoblotting. The intact fusion protein is marked by an arrow. BIP detection is used to confirm the induction of the UPR and TUB detection is used as a loading control. The fold change of seedlings treated with kifunensine (Kif) to seedlings incubated without Kif was calculated after normalization to TUB. Error bars indicate means ± sd (*n* ≥ 3, “ns” not significant, “**” *P* < 0.01, “***” *P* < 0.001 according to Student’s *t* tests).

Finally, we examined the fate of GPI-anchored glycoproteins under conditions that lead to ER stress. Since we were interested in the role of N-glycosylation in this process, we did not apply the commonly used ER stress-inducing agent tunicamycin that causes protein underglycosylation and interferes with glycan-dependent processes in the ER. Instead, we incubated Arabidopsis seedlings for 18 h with dithiothreitol (DTT) that prevents disulfide formation or the proline analog azetidine-2-carboxylic (AZC) acid in the presence or absence of kifunensine. First, we checked whether ERAD is still active under ER stress conditions. In the presence of AZC and kifunensine, degradation of the misfolded glycoprotein ERAD substrate SP-RFP-SUBEX-C57Y lacking a GPI-anchor attachment sequence was blocked. This shows that the glycan-dependent ERAD pathway is still functional when ER stress is induced by AZC (Figure 7A). When incubated with AZC, no apparent stabilization of SP-RFP-LTPG1-C61Y by kifunensine was detected, indicating that it is not subjected to glycan-dependent degradation (Figure 7B). However, when ER stress was induced by DTT, SP-RFP-LTPG1-C61Y levels were significantly increased in the presence of kifunensine (Figure 7C). Apparently, a reducing ER environment interfering with disulfide formation allows degradation by the glycan-dependent ERAD pathway. In summary, these data suggest that under global stress conditions structurally compromised glycoproteins carrying a GPI-anchor attachment sequence are degraded by ERAD.

## Discussion

**Figure 8:**
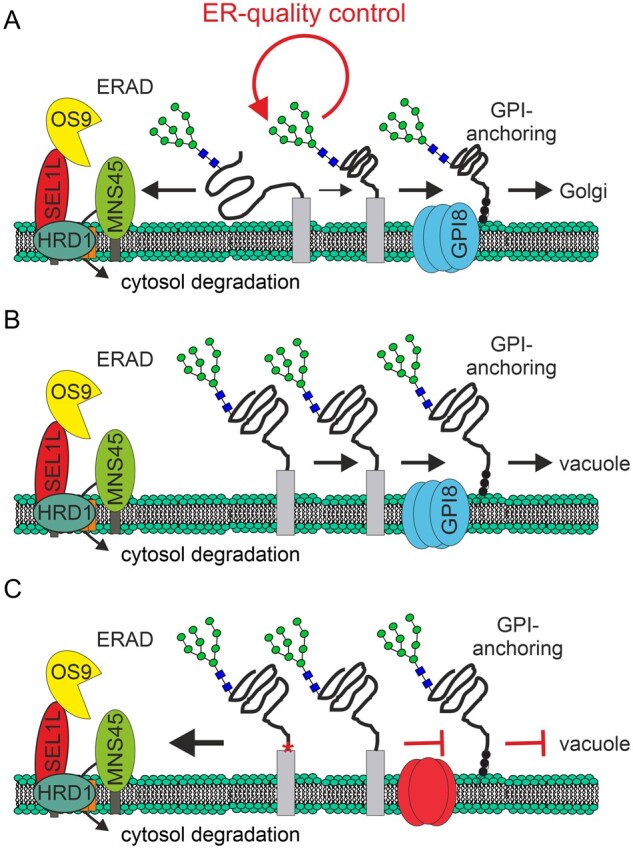
Model for the interplay of ERQC, ERAD, and GPI-anchoring in plants. A, Proteins carrying a GPI-anchor attachment sequence are subjected to ER-quality control processes that monitor their folding status. Severely misfolded proteins are membrane-anchored through their C-terminal hydrophobic GPI-anchor attachment sequence (gray rectangle) and subjected to ERAD if the protein fails to achieve folding. Properly folded proteins, on the other hand, are released from stringent ERQC, GPI-anchored by the GPI transamidase complex (containing the GPI8 subunit) and allowed to exit the ER to the Golgi apparatus. An oligomannosidic N-glycan is indicated as a cartoon (filled green circles: mannose; filled blue squares: GlcNAc residues). B, From non-native proteins with less severe folding defects, the GPI attachment signal peptide is cleaved off by the GPI-transamidase followed by the GPI-anchor attachment. The GPI anchor serves as an efficient ER export signal and the non-native protein is diverted away from ERAD and targeted to the vacuole for degradation. C, When cleavage of the GPI attachment signal peptide is abolished due to a mutation in the omega-site (indicated by a red asterisk) or due to a deficiency in the GPI-transamidase complex (*gpi8-1* mutant), the protein is retained in the ER and subjected to ERAD. The recognition and prolonged interaction with distinct ERQC factors discriminates between severely misfolded proteins and less severely misfolded proteins and sends them to different degradation routes.

The pathways leading to the degradation of different types of misfolded proteins are poorly defined in plants. In the ER, newly synthesized proteins destined for the secretory pathway encounter a number of molecular chaperones and enzymes that assist protein folding, quality control processes and posttranslational modifications such as N-glycosylation or GPI-anchor attachment. While certain glycoproteins are subjected to a single interaction with lectin chaperones, other proteins encounter several binding events to complete their folding (Soldà et al., 2007). Here, we investigated the fate of misfolded secretory glycoproteins carrying a GPI-anchor attachment sequence. Our data show that severely misfolded glycoproteins are efficiently subjected to glycan-dependent degradation as observed previously for soluble and membrane-anchored variants (Su et al., 2011; Hüttner et al., 2014b; Shin et al., 2018). While recent studies from mammals and yeast observed primarily a lysosomal/vacuolar degradation, misfolded GPI-anchored proteins are removed by ERAD with proteasomal clearance in trypanosomes (Ashok and Hegde, 2008; Satpute-Krishnan et al., 2014; Sikorska et al., 2016; Tiengwe et al., 2018). In contrast, our findings indicate that a stringent ER-quality control process prevents the attachment of the GPI anchor to severely misfolded glycoproteins in plants (Figure 8A). Such an early selection for ERAD appears beneficial as it avoids problems that have been associated with the retrotranslocation and subsequent degradation of GPI-anchored proteins carrying a lipid moiety. Moreover, N-glycosylation and N-glycan-dependent quality control processes already occur cotranslationally. The cleavage of the C-terminal peptide, on the other hand, is a posttranslational event after the translocation of the nascent polypeptide chain has been completed (Braakman and Hebert, 2013; Kinoshita and Fujita, 2016). Because of the absence of the GPI-anchor modification, the misfolded protein behaves like other soluble or membrane-anchored ERAD substrates and is efficiently cleared by the HRD1 ERAD complex (Hüttner et al., 2014b; Shin et al., 2018).

The ERAD substrate SUBEX-C57Y lacking any transmembrane domain is normally detected in the soluble fraction (Shin et al., 2018). We propose that the C-terminal GPI-anchor attachment signal peptide bearing a strongly hydrophobic sequence stretch is not cleaved and serves as a transmembrane domain, because we detected the protein primarily in the membrane fraction. Recently, we identified the GPI anchor on purified SP-RFP-COB1-C-term by MS (Beihammer et al., 2020). When we used the same procedure to purify SP-RFP-SUBEX-C57Y-COB1-C-term and analyzed the digested protein by liquid chromatography–electrospray ionization-MS, it was not possible to identify peptides corresponding to the intact or processed C-terminus. A possible reason is the hydrophobic nature of the C-terminal peptide that presents an obstacle for its MS-based detection. This is in agreement with our finding that C-terminally unprocessed proteins could only be solubilized by detergent.

CNX and α-glucosidases involved in early trimming of the transferred N-glycan in the ER have been identified as factors that affect GPI-inositol deacylation in mammalian cells (Liu et al., 2018). Impaired deacylation results in reduced sorting to ER exit sites and ER-to-Golgi transport of GPI-anchored proteins (Tanaka et al., 2004; Fujita et al., 2011). The GPI-anchor remodeling is required for efficient interaction with the p24 family proteins that act as cargo receptors for recruitment of GPI-anchored proteins into COPII transport vesicles. A recent study showed that p24 family proteins are involved in transport of GPI-anchored proteins in plants (Bernat-Silvestre et al., 2020). The functions of ERQC, N-glycan processing and GPI-anchor remodeling for p24 protein interaction and plasma membrane transport in plants remain to be shown. In mammalian cells, most GPI-anchored proteins are N-glycosylated and the CNX/calreticulin cycle is required for proper folding, likely by prolonging ER retention to enable efficient GPI-inositol deacylation (Guo et al., 2020). In addition, there seems to be a yet incompletely understood link between ERAD and GPI-biosynthesis regulation observed in GPI-transamidase-deficient mammalian cells that further highlights the tight interplay of these pathways in the ER (Wang et al., 2020).

It is of note that aberrant proteins such as LTPG1-C61Y lacking severely misfolded ERAD domains are not efficiently degraded by ERAD, but instead are GPI-anchored and targeted to the vacuole (Figure 8B). This shows that ERQC processes distinguish between different types of misfolded substrates and send them to alternative routes for degradation. While all the tested misfolded proteins are N-glycosylated and the mutations affect conserved cysteine residues, other yet unknown features determine the fate of the aberrant proteins. The association with CNX/calreticulin is potentially required for the folding of GPI-anchored proteins, GPI-anchor modifications, and regulation of the ER retention time. Notably, in mammalian cells misfolded GPI-anchored proteins trafficking from the ER to the plasma membrane and finally to the lysosome have been found at the plasma membrane in a complex with CNX and other ER-resident factors (Zavodszky and Hegde, 2019). This indicates that other factors than the release from CNX/calreticulin may be involved in the regulation of ER retention and quality control processes. In plants, GPI-anchored proteins are likely also internalized from the plasma membrane and end up in the vacuole as part of their normal turnover. Whether some aberrant GPI-anchored proteins are targeted to the plasma membrane prior to vacuolar degradation remains to be shown. Our data for LTPG1-C61Y and LTPG1-C116Y, however, favor a direct transport route from the ER to the vacuole without trafficking to the Golgi apparatus.

The GPI anchor presents an efficient ER exit signal in plants (Xue et al., 2017; Bernat-Silvestre et al., 2020). In GPI-transamidase-compromised mutant plants (e.g. the *gpi8-1* mutant) or when the GPI-anchor cleavage site is mutated, less protein is GPI-anchored and the ER retention time is prolonged, which shifts the balance towards degradation by ERAD (Figure 8C). This dynamic routing of misfolded GPI-anchored proteins to ERAD or other degradation pathways has been observed in yeast mutants lacking different degradation pathways (Sikorska et al., 2016). In HRD1-deficient cells, increased targeting to the vacuole was observed and in p24-deficient cells, the majority of misfolded GPI-anchored protein turnover was HRD1-dependent. For the fraction of misfolded GPI-anchored protein sent to HRD1-dependent ERAD, it was proposed that the GPI anchor is removed by an unknown cellular factor to allow efficient clearance by ERAD (Sikorska et al., 2016). This factor has not been identified yet. In light of our findings, it will be interesting to follow the fate of misfolded GPI-anchor attachment sequence-containing proteins in the recently described Arabidopsis p24 delta family mutant (Bernat-Silvestre et al., 2020). In cells lacking a functional p24 cargo receptor complex, aberrant proteins like LTPG1-C61Y might be GPI-anchored and retained in the ER.

Different processes including ERAD and autophagy are involved in clearance of misfolded proteins from the secretory pathway (Liu et al., 2012). Mammalian cells have an additional stress-induced process (termed RESET) for rapid export of misfolded ER-retained GPI-anchored proteins to the secretory pathway for subsequent lysosomal degradation (Satpute-Krishnan et al., 2014). Our assay did not allow assessing the fast response to ER stress and we cannot rule out the existence of a RESET-like response in plants. However, in our assay 18 h after induction of ER stress, ERAD of misfolded glycoproteins was detectable, suggesting that ERAD contributes to clearance of misfolded glycoproteins under ER stress. We observed that unfolded protein response is triggered after 18 h of stress treatment and protein levels of folded or misfolded GPI-anchored proteins are generally reduced. The reduced protein levels could result from enhanced clearance from the ER or unfolded protein response (UPR)-induced attenuation of translation or translocation into the ER (Kang et al., 2006).

In summary, our study is consistent with findings that GPI-anchored proteins are poor ERAD substrates and that the GPI anchor provides an efficient ER-exit signal. Compared to mammals, yeast and trypanosomes, it seems that ERQC and ERAD are more stringent in plants when glycoproteins carry a severely misfolded domain. Aberrant GPI-anchored proteins with less severe structural defects, on the other hand, are sent to the vacuole, probably without trafficking trough the Golgi apparatus. The characterized misfolded glycoproteins and their alternative fate make it possible to unravel the underlying mechanisms of competing processes in the ER in future studies.

## Materials and methods

### Plant material

*Arabidopsis thaliana* plants were grown under long-day conditions (16-h light/8-h dark) at 22°C. The *os9, sel1l, mns45*, and *gpi8-1* mutants (R42Q exchange in GPI8) were described previously (Hüttner et al., 2012, 2014a; Bundy et al., 2016). Transgenic plants were generated by floral dipping and subsequent selection of seedlings on hygromycin-containing 0.5× Murashige and Skoog medium. For treatment with kifunensine, 7- to 12-d-old seedlings were incubated for 24 h in 0.5× MS medium supplemented with 1% (w/v) sucrose and 50-µM kifunensine (Santa Cruz Biotechnology). For the induction of ER stress, seedlings were incubated 18 h in 5 mM AZC (Sigma-Aldrich) or 2 mM DTT (Sigma-Aldrich). CHX treatment was done with 12-d-old Arabidopsis seedlings by adding 100 µg/mL CHX (Sigma-Aldrich). *Nicotiana benthamiana* plants were grown on soil under long-day conditions at 25°C. For inhibitor treatments in *N. benthamiana*, 50-µM kifunensine was infiltrated into leaves together with *Agrobacterium tumefaciens* carrying plasmids for transient expression of proteins.

### Plasmid construction

To generate p64, the GPI-anchor attachment sequence from COBRA1 (Schindelman et al., 2001; 31 C-terminal amino acids) was amplified from Arabidopsis genomic DNA with primers COB1_5F/_6R (Supplemental Table S1), digested with *Bam*HI/*Xho*I and cloned into *Bam*HI/*Sal*I digested p47 (Hüttner et al., 2014a). To generate p64-mRFP (SP-RFP-COB1-C-term expression) a PCR product coding for mRFP fused to a signal peptide was amplified from p117 (Shin et al., 2018) with primers CNX1_12F/mRFP-21R, *Spe*I/*Bgl*II digested and cloned into the *Xba*I/*Bam*HI site of p64. The p64-mRFP-SUBEX-C57Y (SP-RFP-SUBEX-C57Y-COB1-C-term) expression vector was generated by PCR amplification of SP-RFP-SUBEX-C57Y using primers CNX1_12F/SUB_16R. To generate the SP-RFP-LTPG1 expression vector, the LTPG1 sequence coding for amino acids 23–193 was amplified from Arabidopsis cDNA with primers LTPG1_1F/_2R and cloned into the *Bam*HI/*Sal*I site of p47 and the RFP fragment was subsequently inserted to generate p76-mRFP. To generate the expression vectors for mutant variants LTPG1-C61Y, LTPG1-C116Y, LTPG1-W, LTPG1-WW, and LTPG1-C61Y-WW, synthetic DNA fragments were ordered from GeneArt (Thermo Fisher Scientific), PCR amplified with primers LTPG1_1F/_2R and cloned into p76. For expression of SP-RFP-NBRI1-5-LTPG1 (p76-mRFP-NBRI1-5), the NBRI1-5 sequence was amplified from *bri1-5* cDNA using the primers BRI1-25F/-26R, *Spe*I digested and cloned into *Xba*I digested p76-mRFP. The constructs for SP-RFP-SUBEX-C57Y-LTPG1-C61Y and SP-RFP-SUBEX-C57Y-LTPG1 expression were generated by replacement of the RFP sequence in p76-mRFP and p76-mRFP-C61Y with SP-RFP-SUBEX-C57Y. SP-RFP-SUBEX-C57Y was amplified by PCR from p117-SUBEX-C57Y with primers CNX1_12F/SUB_16R, *Spe*I/*Bgl*II digested and cloned into *Xba*I/*Bam*HI-digested p76. Expression vectors p117-SP-mRFP-SUBEX-C57Y (SP-RFP-SUBEX-C57Y), p47-SUBEX-C57Y (SP-SUBEX-C57Y-GFP), p113-mRFP (SP-RFP-TMD9), p113-mRFP-SUBEX-C57Y (SP-RFP-SUBEX-C57Y-TMD9), and p31-NbHEXO3 (NbHEXO3-RFP) were all available from previous studies (Shin et al., 2017, 2018). Expression from the p31 expression vector was driven by the CaMV 35S promoter, and expression of the analyzed proteins in all other constructs was driven by the Arabidopsis *UBIQUITIN 10* (*UBQ10*) promoter.

### Confocal microscopy

For subcellular localization studies, the Agrobacterium strain UIA143 carrying one of the different expression constructs or reporters was diluted to an OD_600_ of 0.1 and expressed in leaf epidermal cells of 5-week-old *N. benthamiana* plants following Agrobacterial leaf infiltration as described previously (Schoberer et al., 2009). Confocal images were acquired 2-d post infiltration on an upright SP5 II Confocal Microscope (Leica) using the LAS AF software system (Leica). Samples were excited using a 561-nm laser line for RFP and signals were collected simultaneously from 600 to 630 nm (intensity 10%–30%, gain 100–200). Typically, 1,024 × 1,024 images were collected in 8-bit with 4- to 6-times line averaging and 1 Airy unit in xyz scan mode. Eight-day-old Arabidopsis seedlings were analyzed in the same manner. All used transgenic plants were derived by independent transformation. Two to four independent lines were selected based on immunoblotting with antibodies against RFP and analyzed in the T2 or T3 generation by confocal microscopy. Representative images are shown.

### Immunoblotting

Protein extraction and endoglycosidase digestion with endoglycosidase H (Endo H) and peptide-N-glycosidase F (PNGase F; both from New England Biolabs) were carried out as described (Hüttner et al., 2014b). Primary antibodies against RFP (Chromotek), CNX (Agrisera), binding immunoglobulin protein (BIP; Agrisera), and tubulin (TUB; Sigma-Aldrich) were commercially available. The protein disulfide isomerase antibody was custom-made (Farid et al., 2011). To isolate membrane and soluble fractions, 200-mg seedlings were extracted with 400-µL extraction buffer (100 mM Tris/HCl, pH 7.5, 25% (w/w) sucrose, 5% (v/v) glycerol, 10 mM EDTA, 1 mM DTE). Cell debris was removed by centrifugation at 600*g* for 3 min. The supernatant was centrifuged at 16,000*g* for 1 h. The pellet (M fraction) was extracted in sodium dodecyl sulfate polyacrylamide gel electrophoresis (SDS–PAGE) loading buffer and subjected together with the supernatant (S fraction) to SDS–PAGE and immunoblotting.

### PI-PLC digestion of microsomal fractions

First, 100 mg infiltrated *N. benthamiana* leaves or Arabidopsis seedlings were immersed in liquid nitrogen and homogenized using a mixer mill and steel balls. After addition of 400 µL of extraction buffer (100 mM Tris/HCl, pH 7.5, 25% (w/v) sucrose, 5% (v/v) glycerol, 1% (v/v) protease inhibitor cocktail (Sigma Aldrich)) the samples were centrifuged at 600*g* for 3 min at 4°C. The supernatant was diluted with an equal amount of water and 100 µL of diluted supernatant was centrifuged at 27,000*g* for 90 min at 4°C. After a washing step with 20 mM Tris/HCl pH 7.5, the pellet was resuspended in 20 mM Tris/HCl pH 7.5 and digested with 0.5 U PI-PLC (Thermo Fisher Scientific) by incubation at 37°C for 15 min. Control incubations were made by incubation without PI-PLC. After the incubation, the samples were centrifuged again at 27,000*g* for 60 min at 4°C. The membrane-containing pellet (M fraction) and the supernatant (S fraction) were extracted in SDS–PAGE loading buffer and subjected to SDS–PAGE and immunoblotting.

### GPI-PLD digestion and Triton X-114 phase separation

Since GPI-PLD is not commercially available, we expressed the human GPI-PLD cDNA in HEK293 cells. A synthetic DNA fragment coding for the signal peptide of human GPI-PLD (NM_001503.3) was synthesized by GeneArt. The DNA fragment was cloned into the *Sal*I/*Bam*HI site of gWIZ (Genlantis) to generate gWIZ-PLD-SP. Cell supernatants transfected with gWIZ-PLD-SP express only the GPI-PLD signal peptide and were used as “mock” control. To generate the expression construct for the full-length GPI-PLD, a synthetic DNA fragment coding for GPI-PLD without the signal peptide was synthesized by GeneArt, amplified by PCR with primers PLD_11F/PLD_12R and cloned into *Bam*HI/*Eco*RI digested gWIZ-PLD-SP to generate gWIZ-PLD. Maintenance and transfection of HEK293 cells was done as described recently (Göritzer et al., 2017); 1.5 mL of the cell supernatant was concentrated using Amicon Ultra-0.5 Centrifugal Filter Units (Merck Millipore) to 100 µL.

The preparation of microsomal membrane fractions was essentially the same as described above for PI-PLC digestion with the exception that ultracentrifugation was carried out at 45,000*g*. The supernatant was discarded and the pellet was resuspended in 80 µL reaction buffer (final concentration: 20 mM Tris/HCl pH 7.5, 0.1% (v/v) NP-40, 0.1 mM CaCl_2_, 10 mM NaCl), and incubated for 15 min at 37°C with 20 µL of the concentrated cell supernatant expressing GPI-PLD or the mock construct. In control experiments, 0.5 U PI-PLC were used for the GPI-anchor digestion for 15 min at 37°C. After the incubation, 2 mM EDTA was added and the samples were transferred to ice. For the phase separation, 400-µL buffer (20 mM Tris/HCl pH 7.5, 10 mM NaCl, 2.5% (v/v) pre-condensed Triton X-114) were added to 100 µL of the reaction mixture. Phase separation was carried out by incubation for 30 min on ice, followed by 5 min at 37°C and centrifugation at 45,000*g* for 6 min at room temperature. The detergent phase was washed with buffer containing 0.1% (v/v) Triton X-114 and the phase separation was repeated. The aqueous (A) and the detergent phase (D) were mixed with SDS–PAGE loading buffer and subjected to SDS–PAGE and immunoblotting.

### Statistical analyses

Data are represented by bar charts showing the mean ± sd and all data points. The numbers of repeats (*n*) are provided in each Figure legend. Statistical analysis (Student’s *t* test) was performed using GraphPad Prism 9.0.1.

### Accession numbers

GPI8 (AT1G08750); BRI1 (AT4G39400); COBRA1 (AT5G60920); LTPG1 (AT1G27950); STRUBBELIG (AT1G11130).

## Supplemental data

The following materials are available in the online version of this article.

**Supplemental Figure S1.** Prediction of potential GPI-modification sites in SP-RFP-SUBEX-C57Y-COB1-C-term and SP-RFP-COB1-C-term.

**Supplemental Figure S2.** Subcellular localization in Arabidopsis roots and leaf cells.

**Supplemental Figure S3.** PI-PLC and GPI-PLD digestions of SP-RFP-COB1-C-term and SP-RFP-TDM9.

**Supplemental Figure S4.** SP-RFP-SUBEX-C57Y-LTPG1 is subjected to glycan-dependent ERAD, but not GPI-anchored.

**Supplemental Figure S5.** SP-RFP-LTPG1 and the mutant variant SP-RFP-LTPG1-C61Y are glycosylated at both N-glycosylation sites.

**Supplemental Figure S6.** The cysteine-deficient SP-RFP-LTPG1-C116Y variant is not subjected to glycan-dependent ERAD.

**Supplemental Figure S7.** Prediction of potential GPI-modification sites in LTPG1 and LTPG1-WW.

**Supplemental Figure S8.** SP-RFP-LTPG1-C61Y is not degraded by the glycan-dependent ERAD pathway in Col-0 wild-type plants.

**Supplemental Table S1.** Primers used for cloning of the different expression constructs.

## Supplementary Material

kiab181_Supplementary_Data

## Notes

Y.S. and R.S. conceived and designed the experiments, Y.S. and U.V. performed the experiments, and R.S. wrote the manuscript with input from all authors.

The author responsible for distribution of materials integral to the findings presented in this article in accordance with the policy described in the Instructions for Authors (https://academic.oup.com/plphys/pages/general-instructions) is: Richard Strasser (richard.strasser@boku.ac.at)
