# Supplementary material for: Proper protein folding in the endoplasmic reticulum is required for attachment of a glycosylphosphatidylinositol anchor in plants
Source: Plant Physiol. 2021 Apr 30;186(4):1878–92. doi: 10.1093/plphys/kiab181 (PMC8331152; doi:10.1093/plphys/kiab181)
Supplement: kiab181_Supplementary_Data [file kiab181_supplementary_data.pdf]

Proper protein folding in the endoplasmic reticulum is required for attachment of a GPI-anchor in plants

Yun-Ji Shin, Ulrike Vavra, Richard Strasser

SUPPLEMENTAL MATERIAL

Supplemental Figure S1. Prediction of potential GPI-modification sites in SP-RFP-SUBEX-C57Y-COB1-C-term and SP-RFP-COB1-C-term. (**A**) The amino acid sequence of SP-RFP-SUBEX-C57Y-COB1-C-term is shown. The signal peptide (underlined, SP: 1-26 amino acids) and transmembrane domain region (highlighted in green, 582-602 amino acids) have been predicted by TOPCONS (https://topcons.net/). The potential GPI-modification sites have been predicted by the big-PI Plant Predictor (http://mendel.imp.ac.at/gpi/plant_server.html) and are labelled in red and blue, respectively. The scores of the big-PI prediction are given. (**B**) The amino acid sequence of SP-RFP-COB1-C-term and the result of the big-PI prediction are shown.

Supplemental Figure S2. Subcellular localization in *Arabidopsis* roots and leaf cells. 8-day-old wild-type (Col-0) or mutant (*os9*, *msn45*, *sel1l*) seedlings expressing the indicated SP-RFP-COB1-C-term or SP-RFP-SUBEX-C57Y-COB1-C-term proteins were analysed under the confocal microscope. For kifunensine (Kif) treatment seedlings were incubated for 24 h in 50 µM kifunensine. Scale bar = 10 µm.

Supplemental Figure S3. PI-PLC and GPI-PLD digestions of SP-RFP-COB1-C-term and SP-RFP-TDM9. The proteins were transiently expressed in *N. benthamiana* (*N.b.*), microsomal fractions were solubilized with 0.1% NP-40, PI-PLC or GPI-PLD digested and subjected to Triton X-114 extraction to separate the aqueous (A) and detergent (D) phase. "mock" indicates incubation with a control cell extract expressing a truncated inactive GPI-PLD.

Supplemental Figure S4. SP-RFP-SUBEX-C57Y-LTPG1 is subjected to glycan-dependent ERAD, but not GPI-anchored. (**A**) Schematic illustration of SP-RFP-SUBEX-C57Y-LTPG1. SP: signal peptide; C57Y: amino acid change that causes the misfolding; SUB: SUB domain; LRRs: leucine-rich repeats; PRRs: proline-rich repeats; "Y": N-glycans; GPI: GPI-anchor attachment sequence. (**B**) Confocal images of transiently expressed SP-RFP-SUBEX-C57Y-LTPG1. Images were made 48 hours after infiltration of *N. benthamiana* leaves. Scale bars = 10 µm. (**C**) Endo H digestion and immunoblot analysis of transiently (*N. benthamiana – N.b.*) or stably (*Arabidopsis* Col-0 or *os9* mutant) expressed SP-RFP-SUBEX-C57Y-LTPG1. (**D**) Immunoblot analysis in the presence of kifunensine (Kif). Staining of membranes with Ponceau S (Pon.) or detection of tubulin (TUB) was used as a loading control. (**E**) CHX-treatment of Col-0 and *os9* seedlings. (**F**) Distribution in soluble (S) and membrane (M) fractions of *os9* seedlings. Immunoblot analysis of 0.1% NP-40 solubilized and Triton X-114 phase separated aqueous (A) and detergent (D) fractions from transient expression in *N. benthamiana* (*N.b.*) in the presence of kifunensine.

Supplemental Figure S5. SP-RFP-LTPG1 and the mutant variant SP-RFP-LTPG1-C61Y are glycosylated at both N-glycosylation sites. (**A**) SP-RFP-LTPG1-C61Y was transiently expressed in *N. benthamiana* and subjected to Endo H digestion for 5 min to remove the two N-glycans. The migration position of the "degylcosylated" band (carrying still a GlcNAc residue attached to Asn), the fully glycosylated band and an unspecific band migrating slightly slower than the fully glycosylated form are indicated. The shift in mobility is approximately in the range of 3-5 kDa suggesting the presence of two N-glycans. (**B**) SP-RFP-LTPG1 was transiently expressed in the presence of 50 µM kifunensine (Kif)

to produce oligomannosidic N-glycans that are sensitive to Endo H digestion. SP-RFP-LTPG1 + Kif and SP-RFP-LTPG1-C61Y were subjected to a limited Endo H digestion (1 min with different dilutions of Endo H - 0: no enzyme; 1: 1:375, 2: 1:75, 3: 1:25, 4: 1:5 dilutions of Endo H) followed by immunoblotting to detect partially glycosylated forms. For SP-RFP-LTPG1 and SP-RFP-LTPG1-C61Y the presence of three bands (2 N-glycans, 1 N-glycan, 0 N-glycan) shows that both N-glycosylation sites are used.

Supplemental Figure S6. The cysteine-deficient SP-RFP-LTPG1-C116Y variant is not subjected to glycan-dependent ERAD. (**A**) Schematic illustration of SP-RFP-LTPG1-C116Y. SP: signal peptide; C116Y: amino acid change that causes the misfolding; "Y": N-glycans; GPI: GPI-anchor attachment sequence. (**B**) Confocal images of transiently expressed SP-RFP-LTPG1-C116Y. SP-RFP-LTPG1-C61Y was infiltrated and analysed in the same manner and is shown for comparison. The images were taken 48 hours after infiltration of *N. benthamiana* (*N.b.*) leaves. Scale bar = 10 µm. (**C**) SP-RFP-LTPG1-C116Y was infiltrated into *N. benthamiana* leaves with the indicated OD_{600} of 0.05 and 0.20. Samples were harvested 48 hours after infiltration and subjected to immunoblotting. The arrow indicates the intact fusion protein. Staining of membranes with Ponceau S (Pon.) was used as a loading control. (**D**) Endo H digestion and immunoblot analysis of transiently expressed SP-RFP-LTPG1-C116Y. (**E**) Immunoblot analysis of transiently expressed SP-RFP-LTPG1-C116Y in the presence of kifunensine (Kif). (**F**) Limited Endo H digestion of transiently expressed SP-RFP-LTPG1-C116Y, see Fig. S5 for details.

Supplemental Figure S7. Prediction of potential GPI-modification sites in LTPG1 and LTPG1-WW. (A) The amino acid sequence of *A. thaliana* LTPG1 (AT1G27950) is shown. The signal peptide (underlined, SP: 1-27 amino acids) and transmembrane domain region (highlighted in green, 167-187 amino acids) have been predicted by TOPCONS (https://topcons.net/). The potential GPI-modification sites have been predicted by the big-PI Plant Predictor (http://mendel.imp.ac.at/gpi/plant_server.html) and are labelled in red and orange, respectively. The scores of the big-PI prediction are given. (B) The amino acid sequence of the mutated *A. thaliana* LTPG1-WW is shown. The big-PI Plant Predictor predicted no potential site for this protein.

Supplemental Figure S8. SP-RFP-LTPG1-C61Y is not degraded by the glycan-dependent ERAD pathway in Col-0 wild-type plants. Immunoblot analysis of CHX-treated *Arabidopsis* Col-0 wild-type seedlings expressing SP-RFP-LTPG1-C61Y in the absence or presence of kifunensine (Kif). The arrow marks the full-length fusion protein. The fold change relative to the starting amount of SP-RFP-LTPG1-C61Y was calculated after normalization to tubulin (TUB). Error bars indicate means ± SD (n = 4, the differences at the individual time points are not significant according to a Student's t test).

Supplemental Table S1. Primers used for cloning of the different expression constructs.

A

```
>SP-RFP-SUBEX-C57Y-COB1-C-term (603 amino acids)
MRQRQLFSVFLLLLAFVSFQKLCYCASSEDVIKEFMRFKVRMEGSVNGHEFEIEGEGEGRPYEGTQTAKLKVTKGGPLPFAWD
ILSPQFQYGSKAYVKHPADIPDYLKLSFPEGFKWERVMNFEDGGVVTVTQDSSLQDGEFIYKVKLRGTNFPSDGPVMQKKTMG
WEASTERMYPEDGALKGEIKMRLKLKDGGHYDAEVKTTYMAKKPVQLPGAYKTDIKLDITSHNEDYTIVEQYERAEGRHSTGA
SRAGSVTNLRDVSAINNLYITLGAPSLHHWLAFGGNPYGEKWQGVVCDSSNITEIRIPGMKVGGGLSDTLADFSSIQVMDFSS
NHISGTIPQALPSSIRNLSLSSNRFTGNIPFTLSFLSDLSELSLGSNLLSGEIPDYFQQLSKLTKLDLSSNILEGHLPSSMGD
LASLKILYLQDNKLTGTLDVIEDLFLTDLNVENNLFSGPIPPNLLKIPNFKKDGTPFNTSIITPPPPPVVDPPPATHRAPPVP
RIPPVSGVPPAPFAPFAPLQPQQHPPPSPPLVWSPPSSDNGGGDPWNSVSGQPTLQISPPSGSGSGKFWSTQRRSPFLPNGGS
RSQFSFVAAVLLPLLVFFFFSA
```

Big-PI prediction:

```
potential GPI-modification site was found.
Quality of the site ............... :       P
Sequence position of the omega-site :     578
Score of the best site ............ :   16.49 (PValue = 3.234595e-07)

Potential alternative GPI-modification site was found (second best site).
Quality of the site ............... :       P
Sequence position of the omega-site :     579
Score of the site ................. :   13.04 (PValue = 2.343482e-06)
```

B

```
>SP-RFP-COB1-C-term (286 amino acids)
MRQRQLFSVFLLLLAFVSFQKLCYCASSEDVIKEFMRFKVRMEGSVNGHEFEIEGEGEGRPYEGTQTAKLKVTKGGPLPF
AWDILSPQFQYGSKAYVKHPADIPDYLKLSFPEGFKWERVMNFEDGGVVTVTQDSSLQDGEFIYKVKLRGTNFPSDGPVM
QKKTMGWEASTERMYPEDGALKGEIKMRLKLKDGGHYDAEVKTTYMAKKPVQLPGAYKTDIKLDITSHNEDYTIVEQYER
AEGRHSTGASRAGSRSPFLPNGGSRSQFSFVAAVLLPLLVFFFFSA

Potential GPI-modification site was found.
Quality of the site ............... :       P
Sequence position of the omega-site :     261
Score of the best site ............ :   16.79 (PValue = 2.680103e-07)

Potential alternative GPI-modification site was found (second best site).
Quality of the site ............... :       P
Sequence position of the omega-site :     262
Score of the site ................. :   13.53 (PValue = 1.800156e-06)
```

Supplemental Figure S1. Prediction of potential GPI-modification sites in SP-RFP-SUBEX-C57Y-COB1-C-term and SP-RFP-COB1-C-term. (**A**) The amino acid sequence of SP-RFP-SUBEX-C57Y-COB1-C-term is shown. The signal peptide (underlined, SP: 1-26 amino acids) and transmembrane domain region (highlighted in green, 582-602 amino acids) have been predicted by TOPCONS (https://topcons.net/). The potential GPI-modification sites have been predicted by the big-PI Plant Predictor (http://mendel.imp.ac.at/gpi/plant_server.html) and are labelled in red and blue, respectively. The scores of the big-PI prediction are given. (**B**) The amino acid sequence of SP-RFP-COB1-C-term and the result of the big-PI prediction are shown.

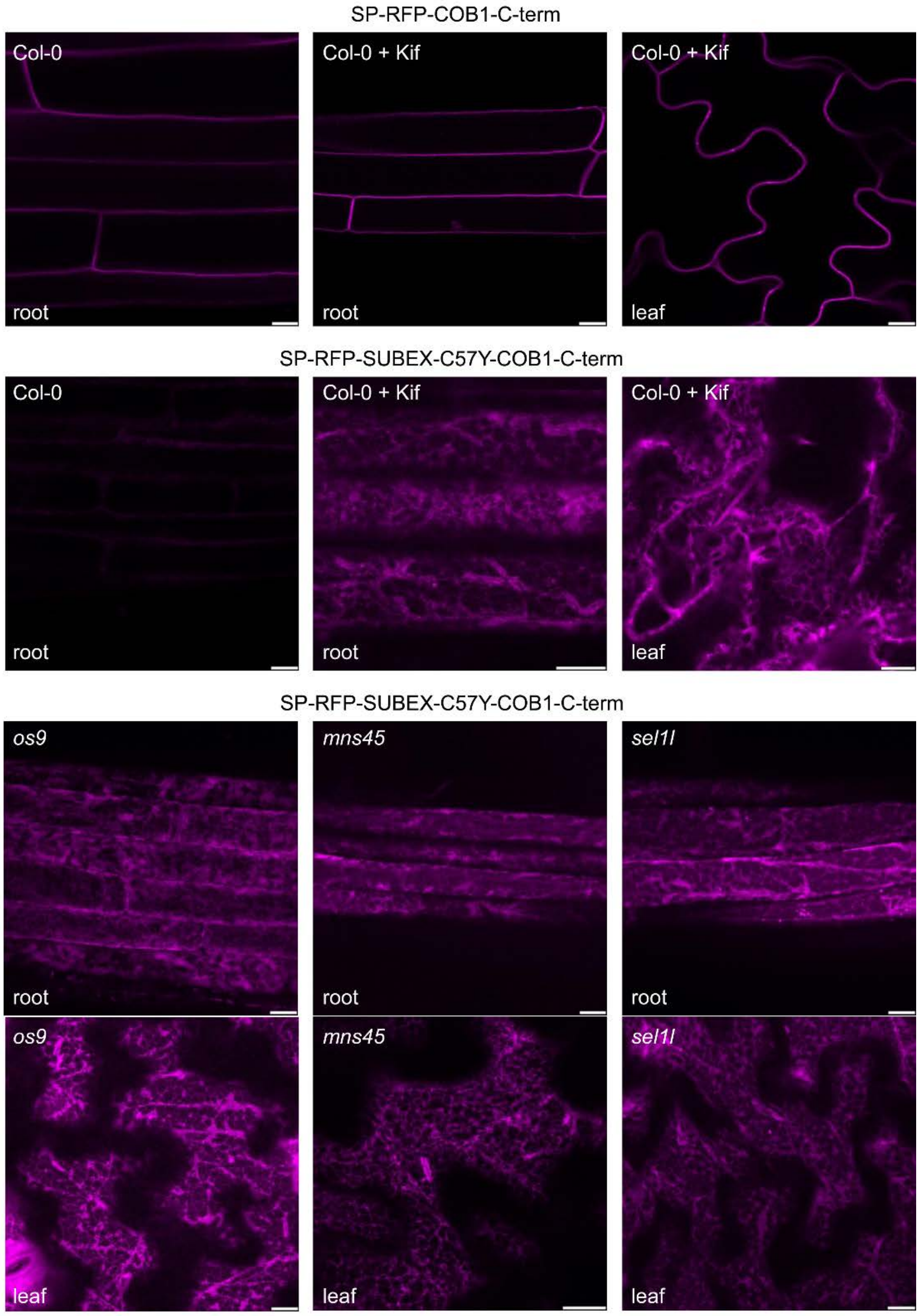

Supplemental Figure S2. Subcellular localization in *Arabidopsis* roots and leaf cells. 8-day-old wild-type (Col-0) or mutant (*os9*, *msn45*, *sel1l*) seedlings expressing the indicated SP-RFP-COB1-C-term or SP-RFP-SUBEX-C57Y-COB1-C-term proteins were analysed under the confocal microscope. For kifunensine (Kif) treatment seedlings were incubated for 24 h in 50 µM kifunensine. Scale bar = 10 µm.

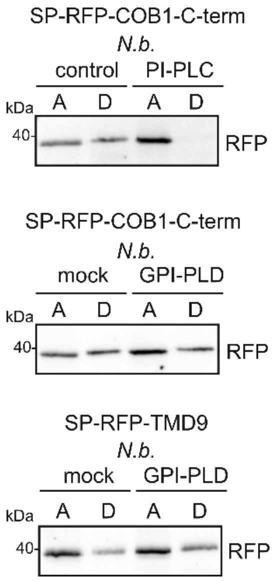

Supplemental Figure S3. PI-PLC and GPI-PLD digestions of SP-RFP-COB1-C-term and SP-RFP-TDM9. The proteins were transiently expressed in *N. benthamiana* (*N.b.*), microsomal fractions were solubilized with 0.1% NP-40, PI-PLC or GPI-PLD digested and subjected to Triton X-114 extraction to separate the aqueous (A) and detergent (D) phase. “mock“ indicates incubation with a control cell extract expressing a truncated inactive GPI-PLD.

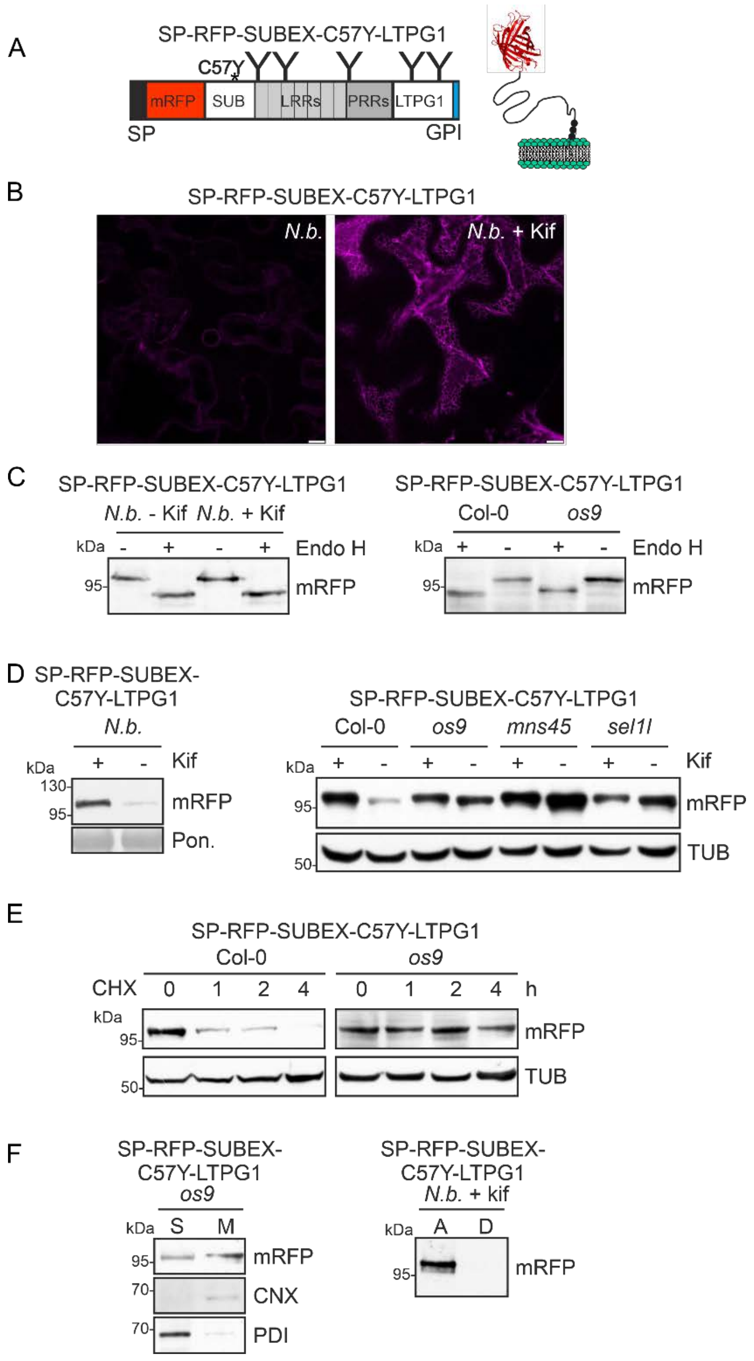

Supplemental Figure S4. SP-RFP-SUBEX-C57Y-LTPG1 is subjected to glycan-dependent ERAD, but not GPI-anchored. (**A**) Schematic illustration of SP-RFP-SUBEX-C57Y-LTPG1. SP: signal peptide; C57Y: amino acid change that causes the misfolding; SUB: SUB domain; LRRs: leucine-rich repeats; PRRs: proline-rich repeats; “Y”: N-glycans; GPI: GPI-anchor attachment sequence. (**B**) Confocal images of transiently expressed SP-RFP-SUBEX-C57Y-LTPG1. Images were made 48 hours after infiltration of *N. benthamiana* leaves. Scale bars = 10 µm. (**C**) Endo H digestion and immunoblot analysis of transiently (*N. benthamiana* – *N.b.*) or stably (*Arabidopsis* Col-0 or *os9* mutant) expressed SP-RFP-SUBEX-C57Y-LTPG1. (**D**) Immunoblot analysis in the presence of kifunensine (Kif). Staining of membranes with Ponceau S (Pon.) or detection of tubulin (TUB) was used as a loading control. (**E**) CHX-treatment of Col-0 and *os9* seedlings. (**F**) Distribution in soluble (S) and membrane (M) fractions of *os9* seedlings. Immunoblot analysis of 0.1% NP-40 solubilized and Triton X-114 phase separated aqueous (A) and detergent (D) fractions from transient expression in *N. benthamiana* (*N.b.*) in the presence of kifunensine.

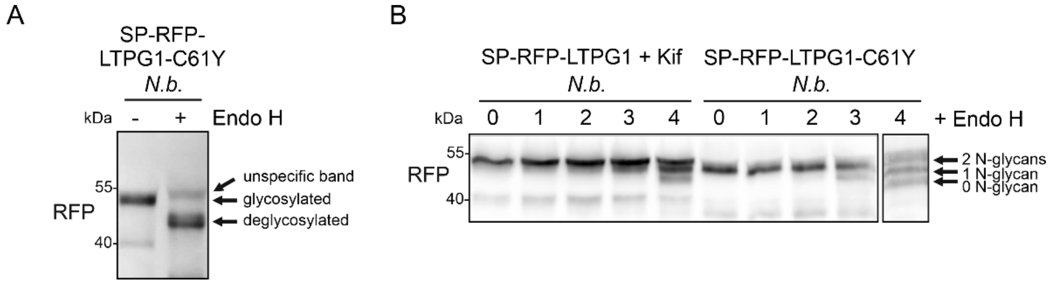

Supplemental Figure S5. SP-RFP-LTPG1 and the mutant variant SP-RFP-LTPG1-C61Y are glycosylated at both N-glycosylation sites. (**A**) SP-RFP-LTPG1-C61Y was transiently expressed in *N. benthamiana* and subjected to Endo H digestion for 5 min to remove the two N-glycans. The migration position of the “degylcosylated” band (carrying still a GlcNAc residue attached to Asn), the fully glycosylated band and an unspecific band migrating slightly slower than the fully glycosylated form are indicated. The shift in mobility is approximately in the range of 3-5 kDa suggesting the presence of two N-glycans. (**B**) SP-RFP-LTPG1 was transiently expressed in the presence of 50 µM kifunensine (Kif) to produce oligomannosidic N-glycans that are sensitive to Endo H digestion. SP-RFP-LTPG1 + Kif and SP-RFP-LTPG1-C61Y were subjected to a limited Endo H digestion (1 min with different dilutions of Endo H - 0: no enzyme; 1: 1:375, 2: 1:75, 3: 1:25, 4: 1:5 dilutions of Endo H) followed by immunoblotting to detect partially glycosylated forms. For SP-RFP-LTPG1 and SP-RFP-LTPG1-C61Y the presence of three bands (2 N-glycans, 1 N-glycan, 0 N-glycan) shows that both N-glycosylation sites are used.

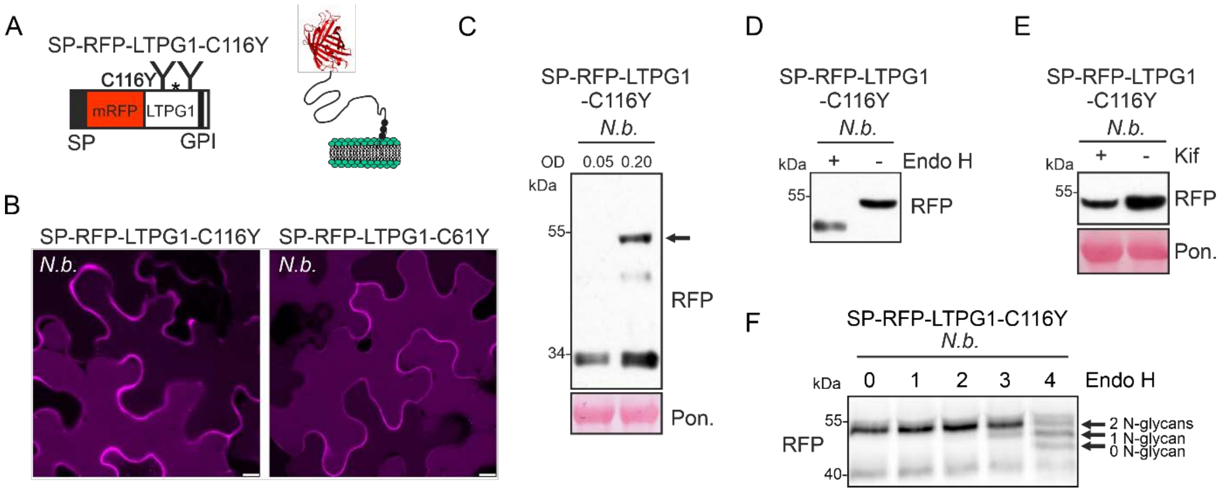

Supplemental Figure S6. The cysteine-deficient SP-RFP-LTPG1-C116Y variant is not subjected to glycan-dependent ERAD. (**A**) Schematic illustration of SP-RFP-LTPG1-C116Y. SP: signal peptide; C116Y: amino acid change that causes the misfolding; "Y": N-glycans; GPI: GPI-anchor attachment sequence. (**B**) Confocal images of transiently expressed SP-RFP-LTPG1-C116Y. SP-RFP-LTPG1-C61Y was infiltrated and analysed in the same manner and is shown for comparison. The images were taken 48 hours after infiltration of *N. benthamiana* (*N.b.*) leaves. Scale bar = 10 µm. (**C**) SP-RFP-LTPG1-C116Y was infiltrated into *N. benthamiana* leaves with the indicated OD_{600} of 0.05 and 0.20. Samples were harvested 48 hours after infiltration and subjected to immunoblotting. The arrow indicates the intact fusion protein. Staining of membranes with Ponceau S (Pon.) was used as a loading control. (**D**) Endo H digestion and immunoblot analysis of transiently expressed SP-RFP-LTPG1-C116Y. (**E**) Immunoblot analysis of transiently expressed SP-RFP-LTPG1-C116Y in the presence of kifunensine (Kif). (**F**) Limited Endo H digestion of transiently expressed SP-RFP-LTPG1-C116Y, see Fig. S5 for details.

A

```
>LTPG1 (length 193 amino acids):
MKGLHLHLVLVTMTIVASIAAAAPAAPGGALADECNQDFQKVTLCLDFATGKATIPSKKCCDAVEDIKERDPKCLCFVIQQAK
TGGQALKDLGVQEDKLIQLPTSCQLHNASITNCPKLLGISPSSPDAAVFTNNATTTPVAPAGKSPATPATSTDKGGSASAKDG
HAVVALAVALMAVSFVLTLPRHVTLGM


Potential GPI-modification site was found.
Quality of the site ............... :        P
Sequence position of the omega-site :      160
Score of the best site ............ :    12.03 (PValue = 3.977965e-06)

Potential alternative GPI-modification site was found (second best site).
Quality of the site ............... :        P
Sequence position of the omega-site :      159
Score of the site ................. :     6.81 (PValue = 4.391470e-05)
```

B

```
>LTPG1-WW (length 193 amino acids):
MKGLHLHLVLVTMTIVASIAAAAPAAPGGALADECNQDFQKVTLCLDFATGKATIPSKKCCDAVEDIKERDPKCLCFVIQQAK
TGGQALKDLGVQEDKLIQLPTSCQLHNASITNCPKLLGISPSSPDAAVFTNNATTTPVAPAGKSPATPATSTDKGWWASAKDG
HAVVALAVALMAVSFVLTLPRHVTLGM

None potential GPI-modification site was found.
```

Supplemental Figure S7. Prediction of potential GPI-modification sites in LTPG1 and LTPG1-WW. (A) The amino acid sequence of *A. thaliana* LTPG1 (AT1G27950) is shown. The signal peptide (underlined, SP: 1-27 amino acids) and transmembrane domain region (highlighted in green, 167-187 amino acids) have been predicted by TOPCONS (https://topcons.net/). The potential GPI-modification sites have been predicted by the big-PI Plant Predictor (http://mendel.imp.ac.at/gpi/plant_server.html) and are labelled in red and orange, respectively. The scores of the big-PI prediction are given. (B) The amino acid sequence of the mutated *A. thaliana* LTPG1-WW is shown. The big-PI Plant Predictor predicted no potential site for this protein.

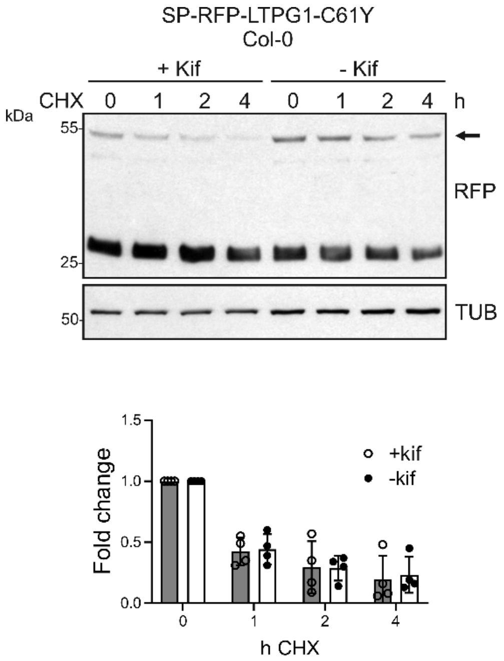

Supplemental Figure S8. SP-RFP-LTPG1-C61Y is not degraded by the glycan-dependent ERAD pathway in Col-0 wild-type plants. Immunoblot analysis of CHX-treated *Arabidopsis* Col-0 wild-type seedlings expressing SP-RFP-LTPG1-C61Y in the absence or presence of kifunensine (Kif). The arrow marks the full-length fusion protein. The fold change relative to the starting amount of SP-RFP-LTPG1-C61Y was calculated after normalization to tubulin (TUB). Error bars indicate means ± SD (n = 4, the differences at the individual time points are not significant according to a Student's t test).

Supplemental Table S1. Primers used for cloning of the different expression constructs.

**Name**	**Sequence (5’ – 3’)**
COB1_5F	TATAGGATCCCCTTTTCTTCCCAACGGTGGTTCC
COB1_6R	TATACTCGAGTTAGGCAGAGAAGAAGAAAAAGAC
CNX_12F	TATAACTAGTATGAGACAACGGCAACTATTTTCC
mRFP-21R	TATAAGATCTAGCTCTAGAAGCACCAGTAGAATG
SUB_16R	TATAAGATCTTCTTTGAGTGGACCAGAATTTTCC
LTPG1_1F	TATAGGATCCGCACCGGCTGCTCCCGGAGGAGC
LTPG1_2R	TATACTCGAGTTACATCCCTAATGTGACATGTCT
BRI1-25F	TATAACTAGTTTTCAAGCTTCACCATCTCAGTCT
BRI1-26R	TATAACTAGTGATTTTGTTTCCGCTAATCGCTAA
PLD_11F	TATAGAATTCCTTCAGCTTCATAATGGTCG
PLD_12R	TATAGGATCCTCACTAATCAGATCCAAGGCTGTACAC
